# Where Microneedle Meets Biomarkers: Futuristic Application for Diagnosing and Monitoring Localized External Organ Diseases

**DOI:** 10.1002/adhm.202202066

**Published:** 2022-12-05

**Authors:** Achmad Himawan, Lalitkumar K. Vora, Andi Dian Permana, Sumarheni Sudir, Airin R. Nurdin, Ririn Nislawati, Rafikah Hasyim, Christopher J. Scott, Ryan F. Donnelly

**Affiliations:** ^1^ School of Pharmacy Queen's University Belfast Belfast BT97BL UK; ^2^ Department of Pharmaceutical Science and Technology Faculty of Pharmacy Hasanuddin University Makassar 90245 Indonesia; ^3^ Department of Pharmacy Faculty of Pharmacy Hasanuddin University Makassar 90245 Indonesia; ^4^ Department of Dermatology and Venereology Faculty of Medicine Hasanuddin University Makassar 90245 Indonesia; ^5^ Hasanuddin University Hospital Hasanuddin University Makassar 90245 Indonesia; ^6^ Department of Ophthalmology Faculty of Medicine Hasanuddin University Makassar 90245 Indonesia; ^7^ Department of Oral Biology Faculty of Dentistry Hasanuddin University Makassar 90245 Indonesia; ^8^ Patrick G Johnson Centre for Cancer Research Queen's University Belfast Belfast BT97BL UK

**Keywords:** biomarkers, diagnosis, external organs, localized diseases, microneedle array patches

## Abstract

Extracellular tissue fluids are interesting biomatrices that have recently attracted scientists' interest. Many significant biomarkers for localized external organ diseases have been isolated from this biofluid. In the diagnostic and disease monitoring context, measuring biochemical entities from the fluids surrounding the diseased tissues may give more important clinical value than measuring them at a systemic level. Despite all these facts, pushing tissue fluid‐based diagnosis and monitoring forward to clinical settings faces one major problem: its accessibility. Most extracellular tissue fluid, such as interstitial fluid (ISF), is abundant but hard to collect, and the currently available technologies are invasive and expensive. This is where novel microneedle technology can help tackle this significant obstacle. The ability of microneedle technology to minimally invasively access tissue fluid‐containing biomarkers will enable ISF and other tissue fluid utilization in the clinical diagnosis and monitoring of localized diseases. This review attempts to present the current pursuit of the application of microneedle systems as a diagnostic and monitoring platform, along with the recent progress of biomarker detection in diagnosing and monitoring localized external organ diseases. Then, the potential use of various microneedles in future clinical diagnostics and monitoring of localized diseases is discussed by presenting the currently studied cases.

## Graphical abstract

This review article brings the current pursuit of external local organ diseases biomarker hunt and the progress of microneedle‐mediated diagnosis and patient monitoring into the spotlight, highlighting the potential development of both fields together to enable ISF‐based diagnosis of these classes of diseases to potentially benefit both patient and healthcare provider.

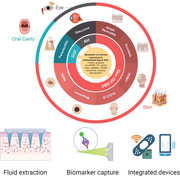

## 1 Introduction

Diagnostic errors are an essential threat to the health care system for all forms of pathological conditions. Misdiagnosis affects the treatment received by the patients, leading to worsened disease or even death and burdening the health system financially.^[^ ^1^, ^2^, ^3^ ^]^ To establish an accurate diagnosis, clinical evaluation, more often than not, needs to be supported by laboratory tests of marker molecules. In the case of nonsystemic diseases, most diagnostic processes rely on visual observation. The problem is that some diseases/conditions do not show any visible change until they reach a particular stage (if at all), preventing an early diagnosis of these illnesses. For example, some skin conditions rely heavily upon clinical scores rather than biochemical markers, leading to an observational bias due to variability in assessment from one clinical professional to another and reducing the consistency in diagnosing the disease.^[^ ^4^ ^]^

Most diseases/pathological conditions, either systemic or local, trigger aberrations in normal human physiological processes rather than maintaining homeostasis. The abnormality can be reflected in the altered concentration of chemical entities, known as biomarkers, in systemic circulation or the affected local tissue and can be either noncausal or casual to the disease incidence or progression. The dynamics of the biomarkers measured can distinguish between typical and pathological conditions. They can also be used to monitor body response or disease prognosis after treatment and interventions.^[^ ^5^ ^]^ Biomarkers can be stratified according to their purposes. Specific molecules can be used: i) to identify the patient's risk of disease (screening biomarker); ii) to identify and confirm the disease in the clinical stage (diagnostic biomarker); iii) to assess the prognosis of diseases (prognosis biomarker); iv) to monitor a disease's severity and clinical responses to treatments (severity biomarker); v) to stratify and determine the correct therapy to a subgroup of patients (predictive biomarker); and vi) to assess pharmacological responses (pharmacodynamic biomarker).^[^ ^6^, ^7^ ^]^

The usefulness of examining biomarkers in disease is a consequence of molecular dysregulation in the diseased cells/tissue leading to changes in molecules, proteins, or metabolites released into systemic and localized circulation. This could include overall levels of the molecule (either up‐ or downregulated), posttranslational modifications, or metabolite profiles. Blood and urine are the most commonly used biomarker sources for diagnosis and can allow a snapshot of biomarker alterations systemically. Both fluids are abundant and can be easily collected for lab measurements. In systemic diseases, this poses no problem other than being considered invasive to take blood samples.^[^ ^8^ ^]^ However, it is essential to highlight that the levels (and changes) of some potential biomarkers of localized pathological conditions are not discernible in the systemic fluid matrix, where it has been established that biomarker differences are effectively lost once they move to the systemic circulation.^[^ ^9^, ^10^ ^]^ In addition, the level of a pharmacologic agent in the blood does not always reflect what it is in the target tissue due to plasma protein binding, making it less convenient for patient monitoring purposes.^[^ ^11^ ^]^

Biomarkers extracted from surrounding ISF may be more valuable for diagnostic or patient monitoring purposes, especially for localized diseases. Generally, as diseased organs or tissues in patients harboring nonsystemic illnesses actively produce metabolites that are unique to pathophysiological processes, the biomarker concentration will be higher in the ailing tissue than in the systemic level counterpart. For example, proteins secreted or shed by tumor cells are several orders of magnitude higher in tumor tissue than in blood.^[^ ^12^ ^]^ Similarly, a pharmaceutical molecule administered to patients will reach the organ target site and bind to its receptor.^[^ ^13^ ^]^ In the case of nonsystemic disease, measuring the concentration of the drug in the target organ can be very useful in predicting the clinical outcome or adverse effects.^[^ ^14^ ^]^

At present, despite its potential usefulness as a rich source of biomarkers, the implementation of ISF for diagnosis is currently underexploited.^[^ ^15^ ^]^ Its poor accessibility mainly causes the problem, and currently, commercially available sampling technologies are not satisfactory.^[^ ^16^ ^]^ Local “tissue liquid biopsy” is an available choice for biomarker harvesting from the tissue in nonsystemic diseases. Nevertheless, it is very invasive, as it requires removing a section of diseased tissue.^[^ ^17^ ^]^ Hence, an innovative way to harvest tissue fluid and biomarkers with minimal invasiveness is needed, and recent microneedle technology is foreseen to fill this technological gap. The microarray patch or microneedle array patch, or MAP for short, is an emerging technology that has been extensively investigated in the field of drug delivery.^[^ ^18^, ^19^, ^20^, ^21^ ^]^ However, it is increasingly being realized that MAPs hold great potential in diagnostics owing to their ability to access extracellular tissue liquid, such as skin interstitial fluid, in a minimally invasive manner. MAPs can be easily applied to exposed tissues or organs, such as the skin, eye or oral cavity, without the aid of expensive equipment or procedures.^[^ ^22^, ^23^, ^24^, ^25^ ^]^ MAPs and their derivative devices might allow biomarker harvesting and sensing from tissue fluid and may help revolutionize the diagnosis and patient monitoring process, especially for local external organ diseases.^[^ ^26^, ^27^, ^28^ ^]^

In the following sections of this review, we will elaborate on the current progress in MAP design for diagnostic and patient monitoring purposes, the status quo of ISF as biomarker reservoirs, the existing and proposed clinical biomarkers for several localized external organ diseases, and the role of MAPs in the research to enable interstitial fluid‐based diagnosis and monitoring of those diseases.

## 2 Interstitial Fluid and Other Tissue Fluids as Biomarker Sources

Extracellular fluid, such as interstitial fluid or aqueous humor, is derived from plasma, so most components mimic the blood composition.^[^ ^29^ ^]^ Moreover, the extracellular liquid is also rich in surrounding cells and tissue secretions, making it a superior option for the clinical diagnosis of localized pathological conditions, as explained in the previous section. A closer look at each biomatrix of interest is discussed below, and highlights of some research regarding biomarker searches in biomatrices of human subjects are compiled in **Table** **1**.

**Table 1:** Summary of research related to extracellular fluid collected from the skin, oral cavity, and eye

Samples	Sample size	Analytical method	Findings	Remarks	Ref.
Skin ISF	Seven healthy human volunteers, four samples were successfully extracted.	Exosome isolation kit + TEM, nanodrop spectrophotometer. Next‐generation sequencing (NGS) for RNA and cDNA. LC–MS/MS for proteomics.	Exosomes and RNA can be found in ISF.	The type of RNA found in ISF is very similar to that in plasma. Overall results demonstrate the diagnosis value of dermal ISF.	[38]
	21 healthy human adult volunteers for the metabolomics study, nine healthy human adult volunteers for the pharmacokinetic study, and 15 type‐I diabetic children and young adults for glucose monitoring.	LS‐MS for high‐resolution metabolomics, caffeine ELISA kit and glucose.	Various compounds can be found in ISF, some unique to plasma. Caffeine pharmacokinetics and glucose level matched the plasma level.	A wide range of biomolecules can be found in ISF and plasma, including bilirubin, carnosine, cortisol, creatine, creatinine, homocysteine, uric acid, vitamin A, B1, B5, B6, C, D2, provitamin D3, lumazine, Lumichrome, hypoxanthine, NAD, uridine, xanthine, some fatty acid, and sterol metabolism. Some unique molecules found in ISF include urocanic acid, 4‐guanidinobutanoic acid, succinyl homoserine, tocopherol, inosine *N*‐methyltryptamine, sphingosine, 20‐COOH‐leukotriene B4, stachyose, gluconolactone, fructose‐6‐phosphate, rhamnose, oxalic acid, and some compound from dietary/xenobiotic sources.	[30]
	Three human volunteers.	LC–MS/MS; samples are processed using tryptic digestion, tandem‐mass‐tagged (TMT) labelling, and 2D separation.	ISF proteomic profiles are homogenous across all subjects. Immunoglobulins can be found in dermal ISF with similar composition compared to the plasma profile.	Some ISF proteins include NADPH 1, creatine kinase B‐type, protein S100‐A4, NADP+, etc. All types of immunoglobulins can be found in ISF, while IgG, IgA, IgD and IgE profiles between plasma and ISF are similar, IgM is less profuse in dermal ISF.	[39, 45]
	15 human volunteers.	HPLC for caffeine quantification and glucometer for glucose quantification.	Caffeine can be detected in ISF, and glucose levels in ISF can be differentiated before and after oral glucose intake.	Proof that exogenous molecule, in this case, caffeine as the model, can be found in human ISF, which highlights the significance of ISF in therapeutic monitoring.	[37]
	Eight human volunteers.	Gel electrophoresis and western blot analysis.	ISF can be harvested using a vacuum chamber from microporous created in the skin by sonophoresis. Several proteins can be identified in the samples.	Albumin, vascular growth factor, and stratifin can be identified in the samples.	[40]
Skin SBF	Ten healthy volunteers.	Untargeted high‐resolution metabolomics, plasma from venipuncture as a comparison.	SBF's metabolites comprise many biochemicals, including amino acids, lipid, pesticides, phytochemicals, purine, pyrimidine, biomarkers, carcinogens, etc.	Unique molecules found include inosine, spermidine, urocanic acid, glycylproline, triethanolamine, 3‐methylsulinylpropyl isothiocyanate, and 2,3,4‐trimethyltriacontane.	[47]
	Eight healthy volunteers.	PIERCE protein assay, immune‐depletion, iTRAQ labelling, 2D‐RPRP‐LCMS.	Comparative study between samples, protein depletion, and iTRAQ improved the analysis's depth and provided robust quantification.	Skin related protein found in SBF includes IL‐18, epidermal fatty acid‐binding protein, ezrin, alpha‐enolase, soluble CD163, protein S100‐A7, S100‐A8, retinoic acid receptor responder protein 2, beta‐2‐microglobulin, suprabasin, secreted Ly‐6/upper‐related protein 1, secreted Ly‐6/uPAR‐related protein 2, C‐reactive protein, dermcidin, and dermokine,	[64]
	Seven healthy volunteers.	2‐D‐HPLC MS/MS after protein depletion.	Approximately 401 proteins were detected in SBF, while only 240 were detected in serum. Proteins found in SBF may be due to cell leaked and related to the cell‐mediated process.	Thirty‐four proteins of interest may be used as biomarkers for local and systemic disease. Beta‐2‐microglobulin, CRP, S100‐A7 protein, clusterin, cystatin A, ezrin, GST Pi, high‐mobility group box 1, IL‐18, IL‐1 receptor antagonist protein, IL‐6 signal transducer, and Thioredoxin are particularly useful as a skin disease biomarker.	[49]
	44 ICU patients (sepsis) and 15 healthy volunteers.	Bio‐Plex 200 System.	The cytokine levels in SBF and serum between healthy subjects and sepsis patients in ICU are differently expressed.	The following cytokine can be found in a detectable amount in SBF: EGF, bFGF, IL‐10, IL‐4, TNF, VEGF, and IL‐6.	[65]
	20 patients with vitiligo and nine healthy volunteers.	ELISA kit.	Comparing cytokines and T‐cell infiltrates between vitiligo and healthy patients. Cytokines can be a valuable biomarker for vitiligo.	CXCL9 and CXCL10 can be found in the blister fluid of affected skin.	[66]
	44 ICU patients (sepsis) and 15 healthy volunteers.	Gelatin zymography and immuno‐fluorometric assay.	Comparing matrix‐metalloproteinase in serum and SBF.	Matrix‐metalloproteinase‐2, ‐8, and ‐9 can be found in SBF.	[67]
GCF	Ten adolescents and ten adults with class I malocclusions and minor upper incisor crowding.	Immunoassay with antibody array kit.	The two age groups show different levels of biomarkers in GCF during orthodontic treatment.	IL‐1, IL‐1RA, MMP‐9, RANKL, and OPG can be detected in GCF.	[68]
	30 subjects were divided into three groups, healthy, gingivitis and periodontitis.	Sandwich enzyme immunoassay.	Vascular endothelial growth factor (VEGF) level in GCF increased as the severity of the disease increased and correlated positively with the VEGF level detected in serum.	GCF collected with the microcapillary method contains a detectable amount of VEFG that may be useful for periodontal disease diagnosis and prognosis.	[69]
	56 subjects with periodontitis and 43 healthy subjects as the control group.	Time‐resolved immune‐fluorometric assay.	*Treponema denticola* infection triggers an increased release of MMP‐8 and MMP‐9 into GCF.	MMP‐8 and MMP‐9 can be detected in GCF collected via intracrevicular washing and can be used as markers for *T. denticola* growth in periodontitis.	[70]
	60 subjects were divided into four groups, healthy group, periodontitis group, type‐II DM group, and periodontitis + type‐II DM group.	ELISA	Resistin level detected in GCF correlates with periodontitis severity and activity, as well as the hyperglycemic state of the patients.	Resistin can be detected in GCF collected with a microcapillary pipette.	[71]
	45 subjects, including a healthy group and a group with periodontal disease.	ELISA	C‐reactive protein can be detected in GCF and correlate with disease severity.	CRP can be detected in GCF collected with a microcapillary pipette. CRP can help assess periodontitis prognosis.	[72]
	28 adult volunteers, 14 of them are healthy, and the rest have a chronic periodontitis.	Capillary electrophoresis samples were derivatized before analysis.	Healthy and chronically ill subjects show different levels of glutamate and arginine. Arginine levels increased in glutamate levels are decreased in subjects with periodontitis.	Arginine and glutamate can be detected in GCF collected via microdialysis and hold diagnostic value for chronic periodontitis.	[73]
	20 subjects with periodontitis, no control group.	HPLC	Ciprofloxacin can be found in GCF at a higher level compared to serum.	A detectable amount of ciprofloxacin can be found in GCF collected with a micropipette. This finding is signaling the possibility of therapeutic monitoring for patients taking oral antibiotics for periodontal disease.	[52]
	32 subjects with periodontitis, no control group	ELISA	The higher IL‐1*β* GCF levels found in periodontitis sites and the presence of three red complex periodontal pathogens and IL‐1B(3954)‐SNP compared with healthy sites within the same individuals.	IL‐1*β* can be detected in GCF. This finding indicates an independent association of both IL‐1B(3954)‐SNP and red complex bacterial species with increased IL‐1*β* levels in GCF of periodontitis sites.	[74]
Aqueous humor	Thirty nondiabetic patients; 15 subjects with immature cataracts and 15 others with mature cataracts.	Spectrophotometric method	The maturity of the disease was linked with substantial imbalances between AH oxidants and antioxidants. The disproportions were decreased SOD, total proteins and CD, as well as increased LLF.	Peroxidation and antioxidant enzyme can be detected in AH extracted with a needle and syringe during operation. The level is associated with cataract maturity and can be a valuable biomarker for cataract.	[29]
	Five subjects underwent cataract surgery.	PCR	miR‐202, miR‐193b, miR‐135a, miR‐365 and miR‐376a are the most abundant microRNA found in the subjects.	microRNA can be detected in AH extracted using needles and syringes.	[61]
	11 diabetic patients without retinopathy, 11 diabetic patients with nonproliferative diabetic retinopathy, and 12 healthy subjects	ELISA	There is a significant upsurge in the protein biomarkers of retinal (macro)glial cell activation in AH. It can be detected not only when diabetic retinopathy is clinically noticeable but also in its subclinical and early clinical phases.	Glial fibrillary acidic protein, aquaporin one, and aquaporin four can be detected in AH collected with a syringe and needle. These proteins can be used for the early detection of retinopathy in diabetic patients.	[75]
	12 patients with PEG, 17 patients with PEX, and 22 controls	ELISA	Clusterin found in AH is a promising robust classifier to distinguish patients with pseudoexfoliation syndrome (PEX) and pseudoexfoliative glaucoma (PEG).	Clusterin can be extracted from patients’ AH collected during cataract surgery in a detectable amount. Clusterin holds an excellent diagnostic value for distinguishing PEG and PEX cases.	[76]
	52 cataractous patients; 49 patients with phakic open‐angle glaucoma (OAG); 24 patients with pseudophakic OAG.	Multiplex bead‐based immunoassay.	The OAG patients with pseudophakic eyes had simultaneous cytokine level rises, which indicates that the AH microenvironment is changed in pseudophakic glaucomatous eyes.	IL‐6, IL‐8, MCO‐1, TNF‐*α*, EGF, PDGF‐AA, PDGF‐AB/BB, and VEGF can be found in AH collected with a needle and syringe before a surgical procedure. IL‐8 and MCP‐1 levels have a high association with pseudophakic status.	[63]
	Ten subjects with POAG and ten healthy human subjects.	LC–MS	Some lipids from sphingolipid and ceramides in AH are unique to healthy volunteers and vice versa.	Lipid species can be detected in AH from donors, and the variation from unique lipid composition between POAG subjects and healthy subjects may serve as a promising biomarker for POAG.	[60]
	96 patients undergoing cataract surgery.	HPLC–MS/MS	Patients who received gatifloxacin ophthalmic gel preparation showed greater mean values of gatifloxacin level in AH than those who received gatifloxacin solution.	Gatifloxacin can be detected in patients’ AH after topical application.	[77]
	Six patients.	HPLC with fluorescence detector.	Detectable amounts of betaxolol after topical application can be found in the AH. HPLC with a fluorescence detector is a suitable method for determining betaxolol concentration in the AH matrix.	Betaxolol can be detected in patients’ AH after topical application.	[78]

### 2.1 Skin Interstitial Fluid

**Figure 1:**
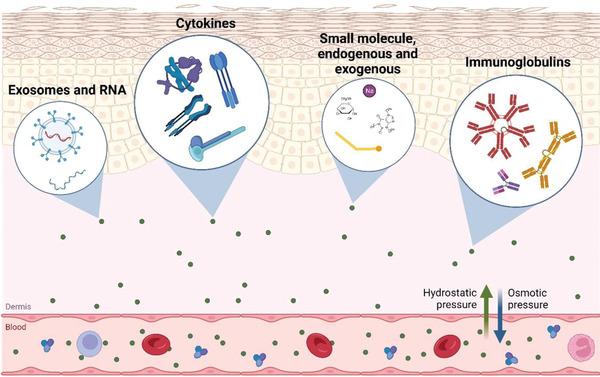
Illustration of biomarker exchange between blood and dermal ISF through hydrostatic and osmotic pressure as well as the types of biomarker class that can be found in dermal ISF, which include, but are not limited to, exosomes and small RNA, cytokines, immunoglobulins and other smaller molecules such as glucose, amino acids, lipids and electrolytes. An illustration is drawn based on the explanation found in various studies.^[^ ^30^, ^31^, ^38^, ^39^, ^45^, ^46^ ^]^

Skin is the most accessible human organ where the ISF is primarily present in the lowermost dermis layer, which consists of 70% ISF by volume.^[^ ^30^ ^]^ ISF surrounds the skin cells and tissue, which bridges the systemic circulation and the cells. ISF is mainly derived from plasma and is maintained through hydrodynamic and osmotic pressure (as illustrated in **Figure** **1**). Its composition mimics or correlates with blood plasma; hence, molecules found in the circulatory system are abundant in ISF. The significant difference between ISF and blood plasma is in their protein content. Due to the negative net charge of many proteins and selective permeability of the capillary membrane of the circulatory system, most proteins cannot permeate out from the blood vessel, making ISF “protein‐poor” compared to plasma.^[^ ^31^ ^]^ Some ISF constituents identified and quantified in healthy human subjects are glucose, cholesterol, cortisol, lactate, lipids, sodium, potassium, chloride^[^ ^16^ ^]^ and various other metabolites, including biomarkers.

Extracting ISF from the skin is trickier than it seems. Although considered one large organ, skin is not homogenous, and anatomical variation can be found depending on the location. The variances include but are not limited to the thickness of the epidermis and dermis layers,^[^ ^32^, ^33^, ^34^ ^]^ the density of glands and hair follicles,^[^ ^35^, ^36^ ^]^ and mechanical properties.^[^ ^32^ ^]^ Intersubject and intrasubject variances regarding the volume of ISF collected for a study have been reported.^[^ ^30^ ^]^ The majority of human studies involving ISF extraction sample the liquid from the forearms of volunteers.^[^ ^30^, ^37^, ^38^, ^39^, ^40^ ^]^

Clinically, skin ISF is used for continuous glucose monitoring,^[^ ^41^, ^42^ ^]^ dermatological drug bioavailability determination,^[^ ^43^ ^]^ and tumor microenvironment analysis.^[^ ^44^ ^]^ Regardless of the promising diagnostic potential of dermal ISF, a noninvasive method to extract the fluid in a reasonable volume for quantitative analysis remains a challenge. The invasive procedures may alter ISF composition after triggering a localized inflammatory response in the sampled area.^[^ ^38^ ^]^

### 2.2 Suction Blister Fluid

Suction blister fluid (SBF) or suction effusion fluid (SEF) is a fluid collected from a designated skin area with an external vacuum force. This biofluid is similar to ISF but may also contain some intracellular components and inflammatory markers resulting from the collection method.^[^ ^46^, ^47^, ^48^ ^]^ A study qualitatively assessed ISF and SBF composition using hydrophilic interaction chromatography (HILIC) and reverse‐phase C18 liquid chromatography and found that at least 60% of ISF and SBF composition were similar, including molecules found in plasma. Among them, at least 8% of similar biomolecules are exclusive to ISF and SBF. Hence, studies on SBF composition can provide good insight into ISF as a promising alternative for diagnosis and monitoring platforms.^[^ ^30^ ^]^ Research on SBF strengthens the foundation that skin tissue interstitial fluid contains valuable marker molecules. Currently, SBF holds a crucial diagnostic value for diseases such as epidermal necrolysis, scleroderma, and complex regional pain syndrome type 1 (CRPS1).^[^ ^49^ ^]^

### 2.3 Gingival Crevicular Fluid

Gingival crevicular fluid (GCF) is a physiological fluid and an inflammatory exudate originating from the dense blood vessel network in the gingival tissue situated below the layers of epithelial cells of the dentogingival space. The alteration from normal physiological fluid to inflammatory exudates ensues when the tissue becomes unhealthy.^[^ ^50^ ^]^ In the diseased state, molecules such as plasma proteins, enzymes, inflammation‐related proteins, cytokines, and bacterial proteins can be identified in GCF.^[^ ^51^ ^]^ After oral administration, drug molecules can also be found in GCF.^[^ ^52^ ^]^ Research has proposed the importance of biomarkers found in GCF in diagnosing and assessing the progression of periodontal disease. The dilemma is that transudate GCF generally develops after the condition reaches a certain degree of severity. Early detection of biomarkers from oral tissue, such as gingival tissue, may be helpful for a more predictive purpose. Currently, predictive biomarkers can also be harvested from saliva,^[^ ^53^ ^]^ but it is thought that collecting biomarkers from gingival tissue may offer better selectivity and sensitivity.

### 2.4 Aqueous Humor

Aqueous humor (AH) is a translucent liquid that fills the anterior and posterior chambers of the eye. Its normal clearness allows light to be transmitted through the lens and cornea, making it essential for human optical sensing. AH is a blood surrogate that delivers nutrition, removes metabolites, and carries neurotransmitters. It helps stabilize the eye structure and helps regulate the homeostasis of these avascular ocular tissues. AH permits inflammatory cells and mediators to circulate in the eye in pathological conditions and distribute drugs to different eye structures.^[^ ^54^, ^55^ ^]^ AH is produced by nonpigmented ciliary epithelium. The composition of AH comes from blood plasma through diffusion or ultrafiltration and active secretion of the cell. The latter contributes up to 90% of the overall composition of AH. Factors including local production of metabolites or rapid consumption of specific nutrition by surrounding tissues underwrite the uniqueness of AH composition.^[^ ^56^ ^]^ Human AH contains several electrolytes, proteins, enzymes, lipids, nucleic acids, and exosomes.^[^ ^29^, ^57^, ^58^, ^59^, ^60^, ^61^ ^]^ In addition to its significant physiological importance, AH carries valuable information for ocular disease diagnoses, such as cytokines and growth factors.^[^ ^57^, ^62^, ^63^ ^]^

## 3 Current Methods to Sample Interstitial and Other Tissue Fluids

Localized fluid within the tissue can be present both intra‐ and extracellularly. ISF (and SBF), GFC, and AH are extracellular tissue fluids. Various techniques have been developed to sample those biomatrices properly, but each of them comes with its limitations. A brief discussion of each established method is given below.

**Figure 2:**
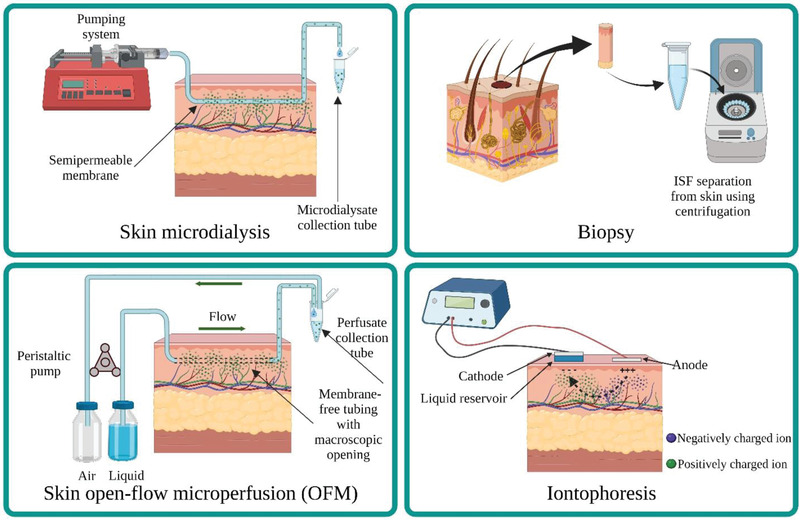
Different dermal ISF extraction methods include microdialysis, open‐flow microperfusion, biopsy, and iontophoresis. The type of molecules and their concentration that can be extracted vary due to the nature of extraction methods, including size limitation, electrical charge limitation or dilution.

The integument, consisting of skin and its derivates, comprising 16% of total body weight, is often considered the largest human organ. Collectively speaking, the skin has the largest liquid available for extraction. Nevertheless, ISF sampling is limited by how minimal the fluid available to extract is for a given area, raising the challenge for its collection. Some techniques have been developed for collecting dermal ISF. These methods include biopsy, microdialysis, open‐flow microperfusion, and reverse iontophoresis (illustration in **Figure** **2**). Each of them is still used in clinical practice, as they are currently the only robust method available for their intended purposes.

Biopsy is the most straightforward method to collect ISF from skin (and from other tissue samples in general). The biopsy terminology is generally understood as a procedure of dissecting a small tissue section for microscopic examination. However, fluid samples can be isolated from this tissue segment. The fluid collection step usually involves centrifugation at high speed. This approach is invasive and very limited for regular ISF extraction. It is generally used for solid tumor samples to monitor their microenvironment^[^ ^12^ ^]^ or to isolate specific biomarkers related to these malignancies.^[^ ^44^ ^]^ Outside its invasiveness, the downside of liquid collection from a biopsy sample concerns cell fluid contamination. At a sufficiently high G‐force (>424 G), the intracellular liquid will start to leak due to the compressive force exerted during tissue centrifugation.^[^ ^44^ ^]^

Microdialysis is a well‐known and widely accepted solute biomarker sampling method for both clinical and experimental settings, particularly for liquid from extracellular spaces.^[^ ^79^, ^80^ ^]^ This technique is initiated by implanting a small probe/catheter with a semipermeable porous membrane into the dermis layer parallel to the skin surface (Figure 2). The fluid pumped through the probe (called perfusate) will reach equilibrium with its surroundings, collect diffusible molecules along the way, and can be pooled at the end of the assembly (dialysate).^[^ ^81^ ^]^ This mechanism allows continuous sampling (and sensing) of the ISF, which is the primary advantage of microdialysis. Skin microdialysis is considered intrusive, as it requires probe insertion in the targeted tissue (in some cases, this procedure might need to be performed under local anesthesia), but the damage caused by this technique is minimal. Another limitation of skin microdialysis is that this technique requires fine‐tuning for optimal extraction and measurement.^[^ ^82^ ^]^

Dermal open‐flow microperfusion (dOFM) is another continuous sampling method proposed as a better alternative for microdialysis. The setup is similar to microdialysis, where probes attached to a perfusate source and an air outlet are implanted under the skin. The machine is operated by a peristaltic pump (Figure 2). dOFM offers a better range of biomarker sampling because dOFM probes are essentially membrane free. This system employs macroscopic fenestrated channels as their probe that enable larger and lipophilic molecules to pass. dOFM sampling devices were reported to be tolerable for an extended time upon testing on human volunteers.^[^ ^83^ ^]^ dOFM assembly is commercially available for human use and is often used to perform a dermal pharmacokinetic study for topical bioequivalence of pharmaceutical preparations.^[^ ^43^ ^]^ The downside of dOFM is that the implantation procedure for its multichannel probes requires anesthesia and could take several hours to perform.^[^ ^30^, ^43^ ^]^

Reverse iontophoretic extraction is probably one of the oldest proposed methods for ISF sampling. To perform this technique, a low electrical current is applied across the skin between two electrodes to increase the permeability of charged and uncharged molecules across the dermis. During this process, ions continuously move across the setup to maintain electrical neutrality.^[^ ^84^ ^]^ Attaching a collection chamber at the end of the electrode may allow the collection and storage of a small volume of ISF.^[^ ^85^ ^]^ Iontophoretic procedures are very limited by the molecule's charge due to its basic principle and the fact that skin itself has a negative net charge, making it selectively permeable for mostly cationic species. It works with small biomolecule extraction, but the instruments are bulky, and the interpretation of the results requires frequent calibration. Although some calibration‐free techniques have already been introduced, these disadvantages still restrict the usage of this procedure for more general purposes.^[^ ^86^ ^]^ Compact iontophoretic wearable devices have emerged to overcome the bulkiness of traditional assembly, but again, the application is mainly limited to small molecule and electrolyte sensing.^[^ ^87^ ^]^

The suction blister technique is an alternative procedure that can extract a sufficiently large volume of skin tissue extracellular fluid faster than previously discussed methods. Liquid extraction is performed by applying sufficient suction force using a vacuum of ≈300–400 mmHg on the skin surface. This procedure, usually aided by elevating the affected tissue temperature, separates the dermis and epidermal layer and creates a small gap. Fluid from the surrounding tissues fills the gap, creating a “blister”, a pocket filled with liquid that can be drawn with a conventional needle and syringe. A good and acceptable SBF specimen must not include blood cells, so the vacuum force applied should not be too high to ensure that the blood capillaries underneath the skin remain intact and retain their “sieve” function.^[^ ^64^ ^]^ The major drawback of this technique is that it triggers an inflammatory reaction upon blister formation that can cause bias when assessing inflammation markers from this biomatrix.^[^ ^46^ ^]^

There are a few alternatives to sample the other two biomatrices discussed in this review. Both fluids have established methods that are often used in daily dental and clinical practices. GCF is transudate and can be collected by a simple sampling technique. The most common sampling methods are intracrevicular washing and microcapillary and paper points/strips absorption techniques.^[^ ^88^ ^]^ Paper points are suitable for routine microbiological analysis, while paper strips are good for biomarker uptake in immunological studies.^[^ ^89^ ^]^ GFC extraction is noninvasive, but the fact that transudate GCF generally develops after the condition reaches a certain degree of severity serves as its own drawback. In many cases, an earlier diagnosis could be more beneficial.^[^ ^53^ ^]^

AH can be harvested using a syringe and is usually collected during invasive interventions such as cataract surgery. In most cases, AH can be extracted from patients undergoing surgery and is typically collected before the operation.^[^ ^61^, ^62^ ^]^ Due to the invasive nature of the extraction process, AH extraction is difficult to perform for day‐to‐day analysis. The application of AH for the early detection and prognostic assessment of ocular disease is limited because patients will need repeated unnecessary invasive sampling.^[^ ^57^, ^62^, ^63^, ^90^ ^]^

From our perspective, the current bottleneck of minimally invasive ISF‐based diagnosis and monitoring advancement is the availability of a consistent and robust alternative sampling method. The method should be suitable for frequent sampling and day‐to‐day analysis, as well as offer advantages that allow early diagnosis of certain conditions (such as what current GCF sampling lacks). In this review, we reaffirm the idea of utilizing MAP technology to enable localized external organ disease diagnosis and monitoring. The potency of MAPs as diagnostic and monitoring platforms will be discussed in the next session by elaborating on the progress researchers have achieved.

## 4 Microarray Patch and Its Application to Collect Biomarkers Containing Tissue Fluid

A MAP is a micron‐scale, needle‐like projection attached to a base arranged in an array formation.^[^ ^91^, ^92^, ^93^ ^]^ MAPs have been exhaustively studied in the pharmaceutical field and have proven to enhance delivery through the skin of many classes of drugs. MAPs have many forms and designs grouped into general categories, such as hydrogel‐forming,^[^ ^37^ ^]^ dissolving,^[^ ^94^, ^95^ ^]^ solid,^[^ ^30^ ^]^ porous^[^ ^96^ ^]^ and hollow^[^ ^97^ ^]^ MAPs. MAPs can be designed and used to access extracellular fluid, such as interstitial fluid in the skin or aqueous humor in the eye, to harvest or sense their biomarkers. Generally, due to their micron‐sized, needle‐like structure, MAPs can enable direct access to a tissue's extracellular fluid with minimal disruption of skin functions.^[^ ^37^ ^]^ Applying an MAP array to the surface of the biological membrane can bypass the barrier and create a transport pathway that allows fluid flow.^[^ ^98^ ^]^ The target biomarker in the fluid can travel along a hollow/porous continuous channel, diffuse through a permeable matrix, be captured by the surface probe, or be sensed by electrochemical sensor assembly.^[^ ^99^ ^]^ The micropores created by needle puncture can then be repaired rapidly by the body.^[^ ^100^, ^101^ ^]^

**Figure 3:**
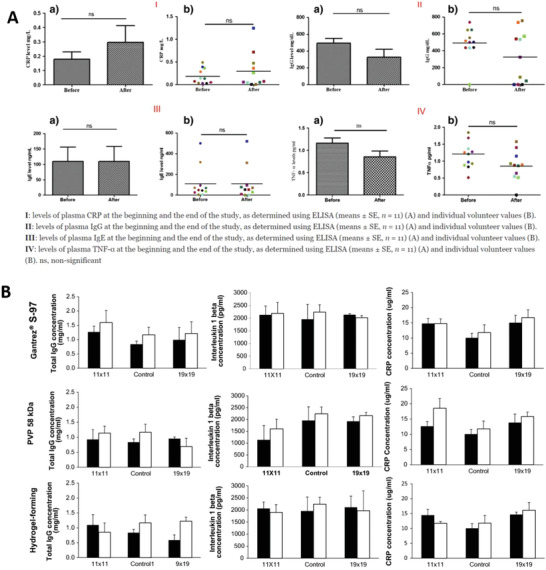
A) The IgG and other serum biomarker levels following the application of hydrogel polymeric MAPs in volunteers’ plasma, showing normal values of IgG, and other biomarkers in humans. Reproduced with permission.^[^ ^103^ ^]^ Copyright 2020, Springer Nature. B) The concentrations of several biomarkers in human serum after the administration of MAPs. White bars and black bars represent values for females and males, respectively (means ± S.D.; *n* = 3). Reproduced with permission.^[^ ^104^ ^]^ Copyright 2017, Elsevier.

In some diseases/conditions, repeated sampling of interstitial fluid may be necessary for prognostic assessment and therapeutic response monitoring. This requirement raises the issue of the biocompatibility of the material used for microneedle array fabrication. More fragile materials possess tip‐breakage risk upon application and may leave residues on the skin, triggering immune and inflammatory responses.^[^ ^102^ ^]^ A suitable minimally invasive microneedle may allow this to be performed without causing discomfort to the patients. Several polymeric‐type MAP arrays have been proven not to trigger any inflammatory or immunologic response after repeated application,^[^ ^103^, ^104^ ^]^ as shown in **Figure** **3**. Hence, when produced with suitable material, MAPs can be a safe and minimally invasive alternative that can advance the utilization of extracellular tissue fluid for diagnosis and patient monitoring in a more widespread clinical setting.^[^ ^105^ ^]^

As mentioned earlier, MAPs can take many forms, depending on the purposes and material used for fabrication. MAP designs varied from a simple solid MAP array to fully integrated wearable devices based on hollow MAPs. From this perspective alone, MAPs can be seen as a versatile technology that offers flexibility ranging from routine sampling in the hospital to wearable devices for continuous monitoring. In this review, the different types of MAPs that have been studied are grouped into three groups primarily based on the complexity of the design and discussed in the following section.

### 4.1 Microneedle Array for Single‐Time Fluid Extraction

**Figure 4:**
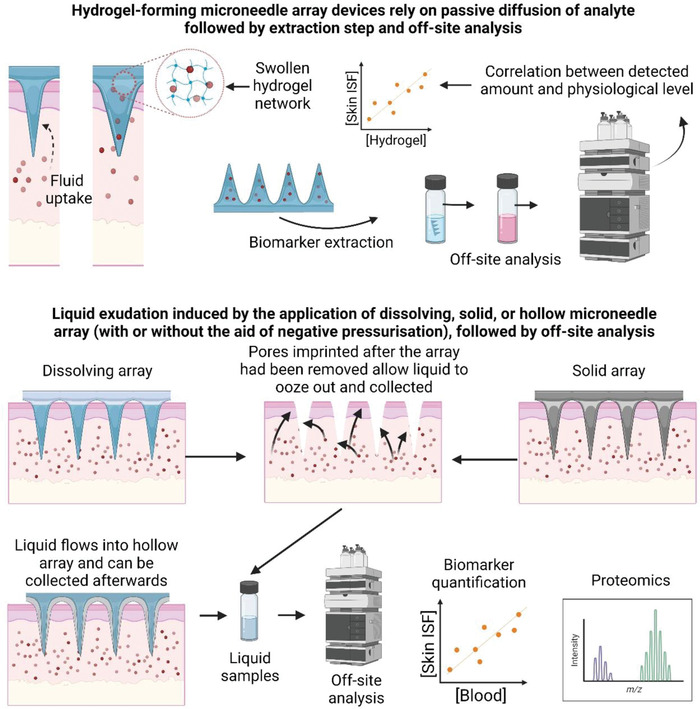
Illustration of different mechanisms of microneedle array devices that can be employed either to nonspecifically capture biomarkers of interest or collect ISF from the tissue for off‐site analysis. The figure is drawn based on the explanation found in various literatures.^[^ ^26^, ^30^, ^37^, ^38^, ^41^, ^95^, ^106^, ^107^, ^108^, ^109^, ^110^, ^111^ ^]^

Single‐time fluid extraction arrays are meant to extract fluid or molecules from tissues for a one‐time application‐extraction‐analysis process. Hollow and porous designs are intended to sample tissue fluid directly, allowing the liquid to flow through its capillary to a reservoir or collection system. Hydrogel‐type MAP arrays rely on a hydrogel swelling mechanism for fluid uptake and marker compound diffusion through their structure rather than direct fluid collection. An illustration is given in **Figure** **4**, and a summary of related research is given in **Table** **2**.

**Table 2:** Research on MAP arrays for one‐time fluid and analyte uptake

Needle type	Material	Liquid uptake	Subject and analyte	Analytical method	Ref.
Swelling hydrogel Array: 19 × 19 Shape: conical NL: 600 µm NB: Ø300 µm	Poly(methyl vinyl ether‐co‐maleic anhydride) (PMVE/MA) and PEG 10 000	NS	Mice; lithium	AAS	[106]
Hollow microneedle Array: 5 × 1 Shape: cylindrical NL: 1500 µm NB: Ø235 µm	Stainless steel (laser cut from 32 G 4 mm needle), glass capillary for collection, and polymeric 3D‐printed housing	Up to 16 µL	Human; exosome, mice; exosome, RNA, protein	TEM, spectro‐photometer, LC–MS/MS for the proteomic	[38]
Swelling hydrogel Array: 19 × 19 Shape: conical NL: 600 µm NB: Ø300 µm	Poly(methyl vinyl ether‐co‐maleic anhydride) (PMVE/MA) and PEG 10 000	NS	Mice; theophylline Human; caffeine, glucose	HPLC, glucometer	[37]
Swelling hydrogel Array: 10 × 10 Shape: pyramidal NL: 1000 µm NB: Ø300 µm	Methacrylate modified hyaluronic acid, photoinitiator, and osmolyte (maltose, sodium chloride, sodium lactate).	3.8 to 7.9 µL	Porcine ear skin (ex vivo); glucose, cholesterol Mice; glucose, insulin	Glucose kit, cholesterol kit, and ELISA	[41]
Swelling hydrogel Array: 10 × 10 Shape: pyramidal NL: 500/800 µm NB: Ø200 µm	Hydrogel I: anhydrous acrylic acid, 2‐hydroxyethyl acrylate, *N*,*N*′‐methylenebisacrylamide, potassium hydroxide, photoinitiator, and additive (hyaluronic acid, dextran, methyl vinyl ether/maleic acid copolymer. Hydrogel II: methyl vinyl ether/maleic acid copolymer, PEG 10 000, and sodium carbonate	3.3 to 6.3 µL	Rats; protein group (from proteomic analysis) including apoC‐I, apoA‐II, Hsc70, HSP90beta, THBP, desmoplakin, SRF1, aFABP, GRP78, ATP1A3, plakoglobin, HSPA1A, lipophilin, DRP‐2, transthyretin, CK1, and CypA.	LC–MS/MS	[26]
Solid, porous, coated microneedle Array: 13 × 13 Shape: pyramidal NL: 1200 µm NB: Ø620 µm	Array: polydimethylsiloxane Coater: hyaluronic acid	0.46 µL min^−1^	Mice; glucose	Glucose paper	[107]
Solid microneedle Array: 5 × 1 Shape: planar NL: 250–650 µm NB: Ø25 µm	Stainless steel 316	Up to 3.4 µL	Human; glucose, caffeine, metabolites	ELISA, glucose analyzer, LC–MS/MS for the metabolomic	[30]
Dissolving microneedle Array: 225 needles over 1 cm^2^ surface Shape: planar NL: ≈500 µm NB: Ø≈275 µm	Needle tip: sodium chondroitin sulfate Backing layer: cellulose acetate and hydroxypropyl cellulose	NS	Rats; vancomycin	LC–MS/MS	[95]
Solid microneedle Array: 305 needles over 50 mm^2^ surface Shape: planar NL: ≈300 µm NB: NS	Polycarbonate	NS	Human; glucose, sodium ion	Microplate reader with fluorescent detector, ion chromatography	[108]
Solid microneedle Array: 305 needles over 50 mm^2^ surface Shape: conical NL: at least 1500 µm NB: NS	Borosilicate glass	1–10 µL	Rats, human; glucose	Glucose test strip	[109]
Solid microneedle Array: 10 × 10 Shape: pyramidal NL: 600 µm NB: 210 µm	Cellulose acetate, polysulfone, polyethersulfone	1.33 mg (mass of ISF)	Mice; glucose	Glucose assay kit	[110]
Solid microneedle Array: 5 × 1 or 9 × 1 array Shape: planar NL: 750 or 650 µm NB: NS	Stainless steel 304, filter paper as the reservoir	>2 µL	Pig skin (ex vivo), rats (in vivo)	NA, only measures liquid uptake	[111]

#### 4.1.1 Solid and Dissolving MAPs

Plain, solid MAPs are probably one of the most straightforward designs of microneedles. Depending on the material, various techniques can be used to produce needle‐like photoetching for metals or molding for softer materials. A solid microneedle array can be manufactured from stainless steel, glass or plastic, or dissolving/biodegradable polymers.^[^ ^112^, ^113^, ^114^ ^]^ The solid needle array can be used to create micropores on the surface of the tissue through which extracellular fluid can seep. An external force such as a vacuum can be used to aid the process and accelerate the fluid flow. When the extracellular liquid flows to the surface, it can be collected using a simple device such as a syringe and needle assembly.^[^ ^30^, ^108^, ^109^ ^]^ Similarly, dissolving MAPs can be used to poke micropores on the tissue surface, and the fluid seeping through the pore can be collected using a pipette.^[^ ^95^ ^]^ The significant advantages are that the liquid uptake does not rely on the MAPs’ capacity to store liquid, the chemistry of the material, or the microfluidic behavior of the structure.

#### 4.1.2 Hollow MAPs

**Figure 5:**
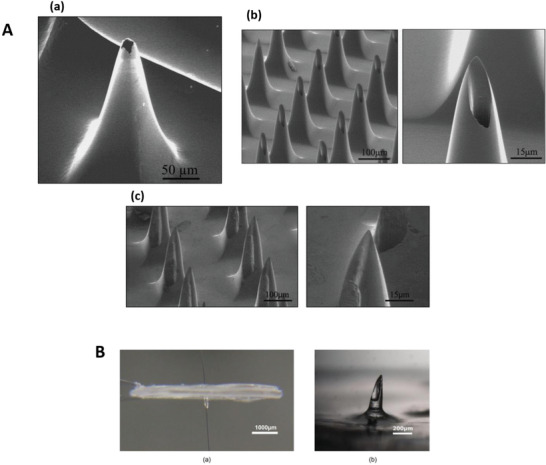
A) SEM images of volcano, hypodermic, and snake Fang microneedle designs. Reproduced with permission.^[^ ^102^ ^]^ Copyright 2004, Elsevier. B) Representative images of polymerized hollow microneedle devices. Reproduced with permission.^[^ ^115^ ^]^ Copyright 2018, Elsevier.

Hollow MAPs can be used to harvest a relatively large volume of ISF from the skin. The extraction volume will depend on the needle length, array size, and time of application.^[^ ^38^ ^]^ The main advantage of the hollow MAP is that it does not require additional extraction steps before analyte quantification.^[^ ^115^ ^]^ Hollow MAPs are usually paired with a collection chamber or channel to accumulate liquid flowing from its microchannel. Hollow MAPs can be produced with lithography, deposition, micromoulding or micromachinery procedures, depending on the needle's construction material^[^ ^102^, ^115^, ^116^, ^117^ ^]^ (**Figure** **5**). Standalone hollow MAPs usually create an open channel that allows the movement of extracellular fluid from the source; therefore, it is possible to use an external force to aid faster fluid flow.

#### 4.1.3 Porous MAPs

As the name suggested, porous MAPs have continuous channels of randomly distributed pores that contribute to large void volume in the solid structure. The advantages of this design are mainly derived from the fast wetting process inside the microchannel and fluid flow driven by capillary action inside it. However, due to the nature of the microstructure, fragility often becomes a hurdle to the further application of porous MAPs.^[^ ^118^, ^119^ ^]^ The porous structure can be created from a hard inorganic material such as alumina particles. Combining inorganic material with polymeric hydrogel or plastic offers some advantages compared to a single inorganic matrix, such as biocompatibility and ease of fabrication.^[^ ^119^ ^]^ The microchannel structure in porous needles created by a relatively simple method, unlike hollow needles, does not require photolithography or other micromachining techniques.^[^ ^96^ ^]^ The surface of the polymer can be modified with chemicals to increase its hydrophilicity, which can help improve fluid flow and uptake.^[^ ^120^ ^]^

#### 4.1.4 Hydrogel‐Forming MAPs

**Figure 6:**
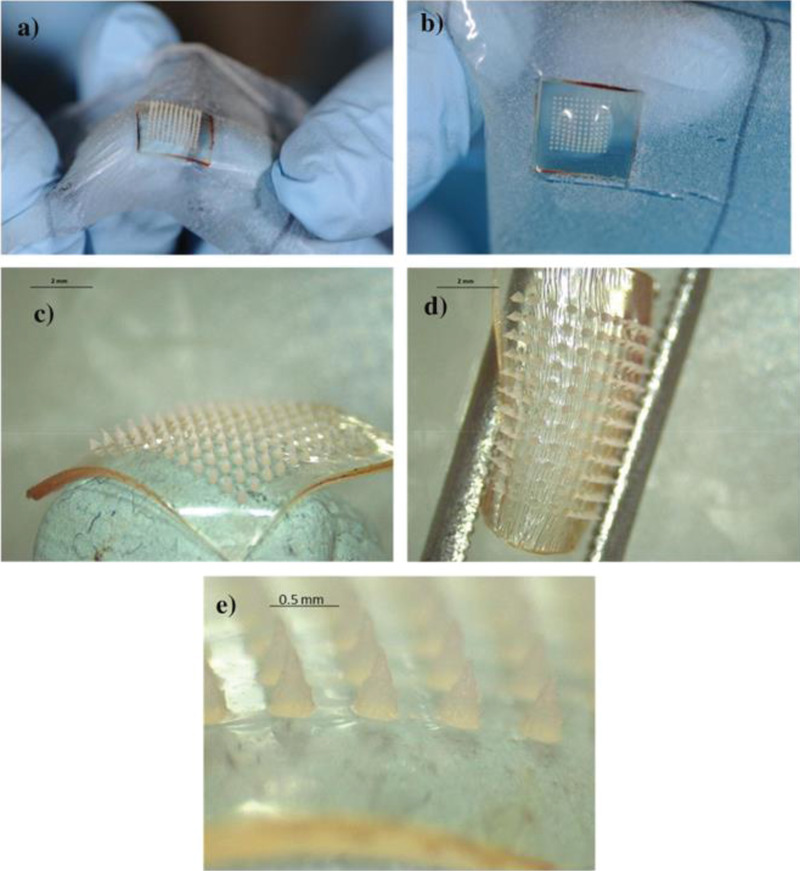
A–D) Photograph of hydrogel‐forming MAPs following application in human volunteers. MAPs were found to be flexible and swollen. E) Importantly, the needles did not change following insertion into the skin. Reproduced with permission.^[^ ^103^ ^]^ Copyright 2020, Springer Nature.

Hydrogel‐forming MAPs are made from polymeric material capable of forming hydrogel structures upon contact with water. After tissue insertion, the microneedles quickly swell through the uptake of available extracellular fluid in the tissue underneath^[^ ^103^, ^121^ ^]^ (**Figure** **6**). When the fluid travels through the “continuous microconduit” formed by the expanded gel network, molecules are diffused through the gel network and retained there. The chemistry of the hydrogel will dictate the magnitude of the diffused molecules and will vary between hydrogels. This is a major limiting factor for HF‐MAPs, as the extracted markers can be different from each hydrogel formulation. This has been demonstrated by a proteome analysis comparing two different swelling hydrogel formulations.^[^ ^26^ ^]^ For an ex situ measurement, an extraction process is needed to pull out analyte molecules from the gel structure. The extraction process can involve either washing the analyte from the hydrogel surface^[^ ^26^ ^]^ or pulling the analyte out from the hydrogel,^[^ ^106^ ^]^ which often does not yield 100% analyte recovery. Here, a calibration is required to determine the relationship between analytes recovered from the hydrogel and the actual physiological concentration.

### 4.2 Microneedle Array Patch for Specific Molecule Capture

**Figure 7:**
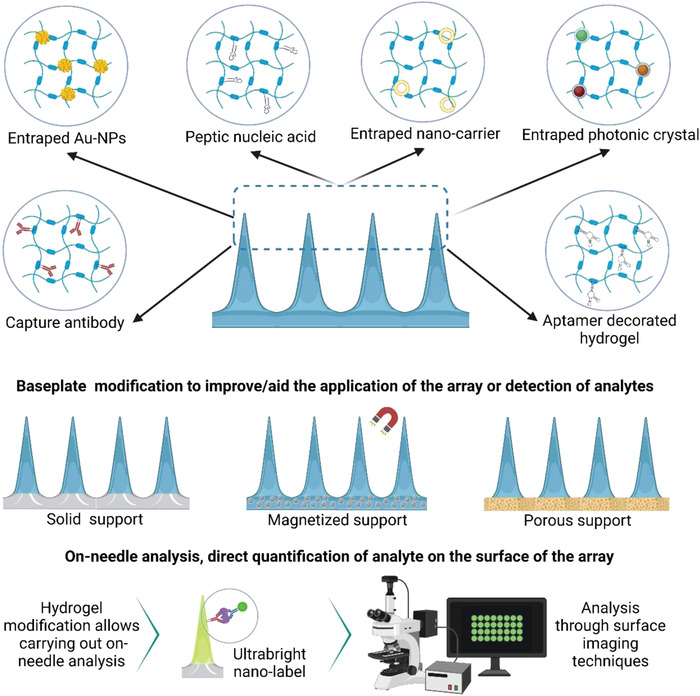
Illustration of different approaches to hydrogel functionalization and modification to enhance capture selectivity and detection sensitivity. The figure is drawn based on the explanation found in various studies.^[^ ^122^, ^123^, ^124^, ^125^, ^126^, ^127^, ^128^ ^]^

Due to its attractive properties and application, the capture and quantification mechanism of biomarker molecules from dermal ISF has specific clinical interest. Biomarker capture technologies are minimally invasive and simple sampling mechanisms that can access abundant, either circulatory or local tissue, biomarkers in the underlying dermal layer.^[^ ^122^ ^]^ In addition, specific analyte capture to the MAPs might allow on‐needle analysis after needle application to the skin.^[^ ^123^ ^]^ These utilities may overcome limitations such as low analyte volume, poor analyte extraction, and dilution by extraction media. An illustration of various types of modification is given in **Figure** **7**, and a collection of progress on the related research is given in **Table** **3**.

**Table 3:** Research on MAP arrays for analyte‐specific uptake and detection

Needle type and dimension	Material	Capture probe	Subject and analyte	Analytical method	Ref.
Solid bilayer, coated Array: 11 × 11 Shape: conical NL: 600 µm NB: Ø300 µm	Array: polystyrene Backing layer: magnetized polystyrene (containing Fe_3_O_4_ nanoparticle) Coater: BSA conjugate	BSA‐cocaine conjugate Capture antibody for IL‐6 Capture antibody for periostin	Mice; cocaine antibody, IL‐6 and periostin. Each analyte was sampled with different coated‐microneedle.	Plasmonic fluor‐linked immunosorbent assay (FLISA). AuNR coated with 800CW fluorophore is used as the plasmonic fluor	[123]
Solid‐supported hydrogel, coated Array: 7 × 7 mm with 77 needles. NH: 550 µm	Array: Poly‐L‐lactide Coater: Alginate‐probe conjugate	Peptic nucleic acid	Human abdominal skin (ex vivo); miR‐210	Fluorescent scanner, quantification based on image intensity. All model DNA were fluorescent‐tagged.	[125]
Solid‐supported hydrogel, coated	Array: Poly‐L‐lactide Coater: Alginate	Physically entrapped stimulatory antigen nanocapsule	Mice; tissue‐resident immune cells	Flow cytometry, cells were stained with anti‐mouse antibody and peptide‐MHC.	[128]
Swelling microneedle, encoded	Poly(ethylene glycol) diacrylate, PEG, 2‐hydroxy‐2‐methyl‐propiophenone	Photonic crystal decorated with antibody probe (TNF‐*α*, IL‐1*β*, and IL‐6 antibody)	Mice; TNF‐*α*, IL‐1*β*, and IL‐6	Spectrometer and a luminoscope.	[122]
Solid, coated microneedle Array: NS, 35 needle Shape: conical NL: 1000 µm NB: Ø150 µm	Polylactic acid	Capture antibody	Mouse skin (ex vivo); IL‐6 and IL‐1*α*	UV–vis spectrophotometry and blotting + densitometry	[126]
Swelling microneedle,	Poly(chondroitin sulfate‐acrylamido‐2‐methylpropane sulfonic acid); *N*,*N*′‐methylene‐bisacrylamide; ‐hydroxy‐4′‐(2‐hydroxyethoxy)‐2‐methyl‐propiophenone	Gold nanoparticle	Rats; carnitine, acylcarnitines, and similar metabolites	LC‐QTOF‐MS	[124]
Solid, porous, coated Array: 10 × 10 Shape: pyramidal NL: 950 µm NB: Ø430 µm	2‐hydroxy‐2‐methyl‐propiophenone, trimethylolpropane ethoxylate triacrylate	LPS aptamer I	Mice; lipopolysaccharide	Microplate reader	[127]

#### 4.2.1 Functionalized Hydrogel MAPs

**Figure 8:**
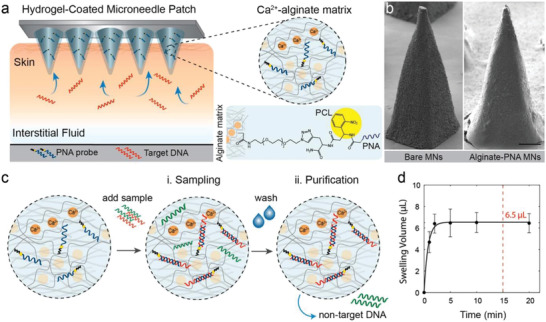
A) Hydrogel MAP arrays prepared from alginate matrix functionalized with PNA probe. B) SEM images of functionalized hydrogel MAPs. C) Representative images of the schematic procedure of the application of this approach for MAP sampling of target biomarkers. D) The swelling kinetic profile of MAPs. Reproduced with permission.^[^ ^125^ ^]^ Copyright 2019, American Chemical Society.

Hydrogel‐forming microneedles can be coated or functionalized with specific probes to capture certain target molecules. The hydrogel can be built on top of a solid needle structure^[^ ^125^, ^128^ ^]^ or cast as a needle array by itself.^[^ ^122^ ^]^ An example of this approach is shown in **Figure** **8**. In this model, fluid uptake in the array is driven by the hydrogel swelling mechanism. Upon swelling, analyte molecules can be captured by the chemically attached probe to the hydrogel network. Depending on the design, captured analytes can be extracted from hydrogels for conventional detection or measured directly on the array (in situ detection) without further separation.

#### 4.2.2 Functionalized Solid MAPs

Solid microneedles can be surface treated and functionalized with a capture probe. Antibody probes are commonly used for this purpose, as they are selective and can be paired with an established immunoassay method. The difference from previously mentioned hydrogels is that this type of needle relies on the abundance of the analyte under the protective tissue layer. As the micropores form after needle insertion, extracellular fluid is expected to flow and fill the small cavities of perforated tissue. The capture probe will attach to the target biomarker after exposure to the liquid. After a particular application time, the needle is removed and washed to collect and pool the analyte in a solvent for detection and quantification.^[^ ^126^ ^]^

#### 4.2.3 Functionalized Porous MAPs

The microchannel surface in the porous needle array structure can be treated to accommodate capture molecule attachment. The main advantage is that the porous structure has a large surface area for contact capture. When the analyte‐rich fluid flows through its continuous cavity, the molecule‐specific analyte is taken up by the surface‐immobilized ligand. As some molecules are captured on the surface of the arrays, on‐needle measurement using quantitative imaging techniques is also possible with this type of array.^[^ ^127^ ^]^

### 4.3 Integrated Microneedle Array for Biosensing and Continuous Monitoring

**Figure 9:**
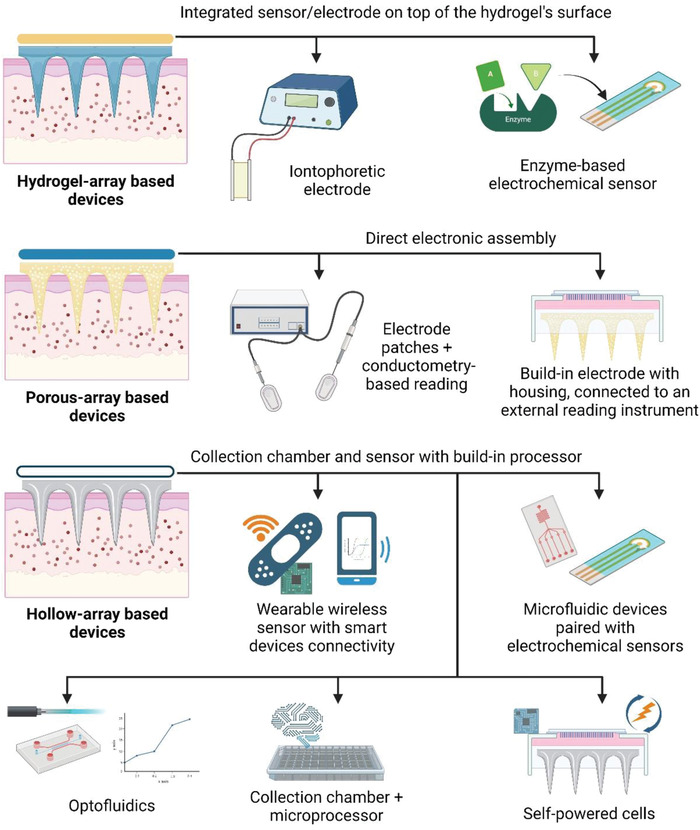
Illustration of different integrations of microneedle array devices for biosensing and continuous monitoring ranging from sensor/electrode‐MAPs integration, incorporation of MAPs into direct electronic assembly, and on‐board analysis prototype where MAP is coupled with collection chamber and build‐in processor/sensor. The figure is drawn based on the explanation found in various studies.^[^ ^27^, ^41^, ^118^, ^129^, ^130^, ^132^, ^133^, ^134^, ^135^ ^]^

MAPs are versatile and can be integrated with various technologies ranging from wearable sensors to continuous monitoring devices.^[^ ^129^, ^130^ ^]^ They allow information gathering, in the form of chemical and biological entities, from previously inaccessible interstitial fluid.^[^ ^131^ ^]^ Integrated MAPs push medical devices to a more modernized, consumer‐friendly technology, and help reduce the need for implantable probes/sensors for more practical usage. An image illustrating different approaches for microneedle array devices with various sensors and instruments is given in **Figure** **9**, and a summary of related studies is given in **Table** **4**.

**Table 4:** Research on integrated microneedle array devices

Needle type and dimension	Material	Integration	Sensor	Subject and analyte	Ref.
Swelling hydrogel needle Array: 10 × 10 Shape: pyramidal NL: 1000 µm NB: Ø300 µm	Methacrylate modified hyaluronic acid, photoinitiator, and osmolyte (maltose, sodium chloride, sodium lactate)	Polyethylene terephthalate substrate (for sensor integration)	Glucose enzymatic electronic sensor	Porcine skin (ex vivo) and agarose gel (in vitro); glucose	[41]
Swelling hydrogel needle Array: 10 × 10 Shape: pyramidal NL: 600 µm NB: Ø300 µm	Polymethyl vinyl ether/maleic anhydride (PMVE/MA) and polyethylene glycol (PEG)	Iontophoretic wearable patch, microfluidic chips	Graphene oxide‐mediated polymerase chain reaction (PCR) and recombinase polymerase amplification (RPA) for signal amplification while the reading was performed using an external device; microfluidic based biosensor.	Mice; Epstein‐Barr Virus (EBV) cell‐free DNA (cfDNA)	[129]
Solid, hollow needle Array: 6 × 6 mm, 200 needle Shape: pyramidal NL: 300 µm NB: Ø50 µm	Silicone	Collection chamber with sensor, potentiostat circuit, and microprocessor.	Screen‐printed, 3‐electrode amperometric sensor, platinum‐carbon (Pt—C) electrode covered with cross‐linked bovine serum albumin containing glucose oxidase.	Glucose	[130]
Solid, hollow needle Array: 3 × 3 Shape: pyramidal, triangular base NL: 800 µm NB: NS NO: 425 µm	Photocurable acrylate‐polymer	Wireless bandage with built‐in sensor	Printed electrode system consisting of Ag/AgCl‐Ecoflex mix and carbon‐based electrode, connected to a polyimide‐based wireless electronic board.	Porcine skin (ex vivo); tyrosinase	[132]
Solid, hollow needle Array: 3 × 3 Shape: pyramidal, triangular base NL: 1500 µm NB: NS NO: 425 µm	Photocurable acrylate‐polymer	Self‐powered biofuel‐cell	Biofuel‐cell of carbon paste electrode transducer	In vitro; glucose	[133]
Solid, porous needle Array: 6 × 6 Shape: conical NL: 100 µm NB: NS	Polydimethylsiloxane and PEG as porogen	Electrolytic assembly	Conductometry	Human; DC electric resistance to monitor edema	[118]
Solid, hollow needle Array: NS Shape: pyramidal, triangular base NL: 1000 µm NB: 500 µm	Eshell 300 polymer	Microfluidic channel and lab‐on‐chip	Electrochemical detector	In vitro, myoglobin and troponin	[27]
Solid, hollow needle, coated	Gold and nickel, thiolated and biotinylated PEG chain, biotin	Optofluidic device, functionalized surface for specific molecule capture	Optofluidic sensor	In vitro; vancomycin	[134]
Solid, porous needle Array: 3 needles on Ø8 mm round base Shape: conical NL: 600 µm NB: NS	Poly‐glycidyl methacrylate	Electrode patch	Ag/AgCl electrodes patch	Human; transepidermal potential	[135]

#### 4.3.1 Hydrogel Needle‐Based Devices

Swollen hydrogel surfaces can interact directly with some sensors owing to their high water content. The additional reactant is usually provided in the interface between the hydrogel and sensory parts of the devices. The wet surface can act as the medium that allows the electrochemical reaction to be triggered and provides reading for the devices.^[^ ^41^ ^]^ The continuous aqueous body provided by the swollen hydrogel can mediate the flow of electric current, bypassing the protective outer layer of tissue. It is feasible to accumulate target analytes or drive liquid movement under tissue surfaces that directly touch the hydrogel and improve the device efficiency, selectivity, and sensitivity.^[^ ^129^ ^]^ Using hydrogels for needle arrays can diminish the need for expensive materials needed for hollow or porous microneedle‐based devices.

#### 4.3.2 Hollow and Porous Needle‐Based Device

The integrated device builds on the bases of hollow or porous needle arrays. Usually, it incorporates a direct sensor into the collection chamber or microfluidic channel to lead the fluid flow into a sensing compartment. Detection of analytes can be made by integrating sensors that can measure the quantity of molecules based on either the optical or electrical dynamics of the fluid.^[^ ^27^, ^134^ ^]^ Human tissue electrical potential, which is one of the indicators of a physiological process, can also be directly measured using this type of integrated gadget.^[^ ^135^ ^]^ Usually, surface functionalization or enzymatic reactions are required to provide better selectivity and sensitivity to the detection process.^[^ ^130^, ^134^ ^]^ Enzymatic‐based detection devices are beneficial for continuous monitoring because the analytes are consumed upon reaction, thus providing the imbalance needed for molecular diffusion of extracellular fluid, assuming the microneedle array acts as a continuous channel that links the liquid in the sensing compartment and the tissue underneath.^[^ ^130^ ^]^ The main advantage of the hollow and porous microneedle array is that they allow direct access to interstitial fluid. Any measurement of the fluid sample with this array is a direct quantification; thus, it does not require calibration to the actual tissue physiological concentration.

## 5 Localized External Organ Disorders and Their Biomarkers of Interest

**Figure 10:**
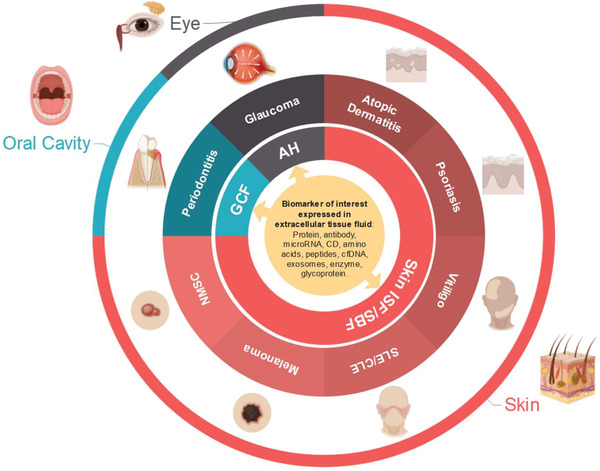
Illustration of selected external organs and their disease, as well as their biomarker source, projected to benefit from microneedle array device‐based diagnosis and patient monitoring. GCF: gingival crevicular fluid; AH: aqueous humor; SLE: systemic lupus erythematosus; CLE: cutaneous lupus erythematosus; ISF: interstitial fluid; SBF: suction blister fluid.

It is mentioned in the earlier section that extracellular tissue fluid‐based diagnosis and patient monitoring are significant obstacles, making it not ready for widespread clinical application. In turn, it also hinders the establishment of relevant biomarkers for many localized external organ diseases. Some of these conditions still rely heavily on visual clinical scoring. However, luckily, scientists did not stop their search for key biomarkers for these diseases. This review emphasizes how external organ disorders might benefit from employing nonblood‐based biomarker detection. We selected several representative pathological conditions in the skin, oral cavity, and eyes and elaborated on their current diagnostic approach. We also point out how far the pursuit of finding their relevant biomarkers is to establish a more reliable diagnosis and monitoring. We put those diseases into a schematic representative, as shown in **Figure** **10**. Individual discussion of each condition is presented in the subsequent sections, and a compilation of biomarkers of interest, both established and proposed for the selected diseases, is provided in Table 1.

### 5.1 Atopic Dermatitis

Atopic dermatitis (AD), also known as atopic eczema, is the most common inflammatory disorder characterized by recurrent eczematous lesions such as erythematous patches, blistering, exudation and crusting that can develop into fissuring, scaling and lichenification in the chronic stage, most commonly with a childhood onset.^[^ ^188^ ^]^ The prevalence of AD is ≈15–20% and can cause significant morbidity that affects the patient's quality of life. Due to this manifestation, patients suffering from AD usually experience sleep disturbances, thus affecting their quality of life.^[^ ^189^, ^190^ ^]^ AD is one of the most common skin diseases worldwide, both in adults (2% to 10%) and children (7% to 30%).^[^ ^191^, ^192^ ^]^ As a heterogeneous disorder, the AD extent of lesions, frequency, and intensity of symptoms vary between individuals.^[^ ^189^ ^]^

The pathophysiology of AD is multifactorial, although recent studies have highlighted that the condition is caused by an immune response that is T‐helper 2 (Th2)‐cell‐mediated and defects of the skin barrier, most notably due to mutation of the filaggrin (FLG) gene. AD diagnosis and treatment depend entirely on clinical scores, which are not prevalent among chronic diseases.^[^ ^4^ ^]^ The unclear mechanism involved in this disorder adds to its complexity of diagnosis. Hence, having a clear and consistent biomarker to objectively assess AD is important and therefore represents a clear unmet clinical need.^[^ ^193^, ^194^ ^]^ Researchers have proposed several biomolecules as markers for AD, including the thymus and activation‐regulated chemokines, macrophage‐derived chemokines, e‐selectin, immunoglobulin E and several other molecules (summarized in **Table** **5**).^[^ ^136^, ^137^, ^138^, ^139^, ^140^, ^141^ ^]^

**Table 5:** Established and proposed biomarkers of selected external organ diseases

Type of disorder (organ)	Biomarker class	Biomarker (expression)	Location	Ref.
Atopic dermatitis (skin)	Protein	^1^Thymus and activation‐regulated chemokine (▲), ^2^macrophage‐derived chemokine (▲), ^3^e‐selectin (▲), ^4^eotaxin‐3 (▲), ^5^edipokines (adiponectin and resistin) (▼)	^1–5^Serum, ^1^endothelial cell	[136, 137, 138, 139, 140]
	Antibody	Immunoglobulin E (▲)	Serum	[141]
Psoriasis (skin)	Protein	^1^Atypical chemokine receptor or 2 chemokine‐binding protein 2 (ACKR2 or CCBP2) (▲), ^2^prokineticin 2 (▲), ^3^calmodulin‐like protein (▼), ^4^TNF‐*α*‐induced protein 3‐interacting protein 1 (▼), ^5^C‐X‐C motif chemokine 10 (♦), ^6^calprotectin (▲, ^7^koebnerisin (▲), ^8^psoriasis‐linked late cornified envelope protein (▲), ^9^cathelicidin (▲)	^1^Cell secretion, ^2–4,8,9^skin cell/tissue, ^5^serum, ^6,7^leukocytes, ^6^epidermal cell, ^7^dermis cell	[142, 143, 144, 145, 146, 147, 148, 149, 150]
	microRNA	miR‐21 (▲), miR‐31 (▲), miR‐135b (▲), miR‐136 (▲), miR‐138 (▲), miR‐146a (▲), miR‐155 (▲), miR‐184 (▲), miR‐203 (▲), miR‐201 (▲), miR‐221/222 (▲), miR‐424 (▲), miR‐99a (▼), miR‐125b (▼), miR‐141 (▼)	Skin	[126, 127]
Vitiligo (skin)	RNA	^1^miRNA‐211 (▼)	^1^Skin	[151]
	Protein	^1^C‐X‐C motif chemokine ligand (CXCL) 9 (▲), ^2^C‐C motif chemokine ligand 20 (CCL20) (▲), ^3^S100 calcium‐binding protein B (S100B) (▲), ^4^C‐X‐C motif chemokine ligand (CXCL) 10 (▲), ^5^Tumour necrosis factor‐*α* (TNF‐*α*) (▲)	^1,2^SBF, ^2–4^skin, ^2^plasma, ^3–5^serum	[66, 152, 153, 154, 155]
	Amino acid	Homocysteine (▲)	Serum	[156]
	Cluster of differentiation	Soluble CD (sCD) 25 and 27 (▲)	Serum	[157]
SLE/CLE (skin)	Protein	^1^MxA protein (♦), ^2^guanylate binding protein‐1 (▲), ^3^complements (▼), ^4^galectin‐9 (▲), ^5^IFN‐*α* (▲)	^1,2^Skin, ^3–5^plasma	[17, 132, 158, 159, 160, 161, 162, 163]
	mRNA	Type I IFN‐regulated genes (▲)	Plasma	[162]
Melanoma (skin)	Protein	Programmed death‐ligand 1 (PD‐L1) (▲), S100A8/A9 protein (▲)	Skin	[164, 165]
	microRNA	^1^Let‐7 family (▲), ^2^miR‐15b (▲), ^3^miR‐199a‐5p (▲)	^1,2^Skin, ^3^serum	[166, 167, 168]
	Circulating tumor DNA	ctDNA (▲, released by tumor cell)	Plasma	[169]
	Enzyme	Lactate dehydrogenase (▲), tyrosinase (▲), cyclooxygenase‐2 (▲), matrix metalloproteinases (▼)	Serum	[17]
	Exosome	PD‐1+/CD28+ Exo (▲)	Serum	[170]
Non‐melanoma skin cancer (skin)	Protein	p53 autoantibody (▲), matrix metalloproteinase‐7 autoantibody (▲), heat shock protein 70 autoantibody (▲)	Serum	[171]
	Enzyme	Nicotinamide *N*‐methyltransferase (▼)	Skin	[172]
	microRNA	^1^miR‐203 (▼), ^2^miR‐21 (▲), ^3^miR‐184 (▲), ^4^miR‐186‐5p (▲), ^5^miR‐145‐5p (▲), ^6^miR‐30a‐5p (▼), ^7^miR‐25‐3p (▼), ^8^miR19a‐3p (▼)	^1–3^Skin, ^4–8^plasma	[173, 174, 175]
Periodontitis (oral)	Protein	^1I^ IL‐1*β* (▲), ^2^TNF‐*α* (▲), ^3^elastase (▲), ^4^IL‐6 (▲), ^5^osteocalcin (▲), ^6^MMP‐8 (▲), ^7^MMP‐9 (▲), ^9^MMP‐13 (▲), ^10^MIP‐1*α* (▲)	^1–10^GCF, ^1,2^tissue	[176, 177, 178, 179, 180]
	Glycoprotein	Oncostatin M (▲)	GCF	[177]
	Peptide	Cross‐linked N‐terminal telopeptide (NTx) of Type I collagen (▲)	GCF, serum, periodontal cavity fluid	[181]
Glaucoma (eye)	Protein	Myocilin (▲)	Aqueous humor	[182]
	Glycoprotein	Autotaxin (▲), endothelial leukocyte adhesion molecule‐1 (▲), CD44 (▲)	Aqueous humor	[183, 184, 185]
	Peptide	N‐terminal fragment of proatrial natriuretic peptide (▲)	Aqueous humor	[186]
	Enzyme	Glutathione peroxidase	Aqueous humor	[187]

Note: (▲) = upregulated expression, (▼) = downregulated expression, and (♦) = varying expression.

### 5.2 Psoriasis

Psoriasis is a chronic papulosquamous inflammatory disease affecting the skin and is considered an autoimmune disease. Psoriasis has a high genetic relationship that can occur at any age, and younger onset is related to a more severe clinical manifestation and complication.^[^ ^195^, ^196^ ^]^ It affects ≈2–3% of the adult population and 0.5–1% of the child population worldwide. The most common clinical presentation of psoriasis, which accounts for 80–90% of all psoriasis cases, is plaque‐type psoriasis characterized by symmetrical, well‐demarcated, erythematous plaques and silvery scales. Patients’ quality of life can be severely impaired due to the visual appearance of the psoriatic area.^[^ ^197^, ^198^, ^199^ ^]^ In addition, up to 30% of patients with psoriasis may develop psoriatic arthritis (PsA), a chronic inflammatory joint disease that involves a complex immunological and inflammatory mechanism.^[^ ^200^ ^]^ Complications of psoriasis can extend beyond cutaneous and joint abnormalities, affecting various organs, such as the cardiovascular system. Psoriasis complications are also known to be associated with diseases such as diabetes, malignancies, anxiety, and depression.^[^ ^197^ ^]^

Psoriasis diagnosis is primarily based on the patient's clinical history, Psoriasis Area and Severity Index (PASI), in conjunction with morphological and histopathological examination of the lesion. The differential diagnosis criteria for this disease have not yet been well defined. This diagnosis issue is particularly problematic because the pathological change in the skin is not observable at the early stage of this disease. Psoriasis pathophysiology is multifactorial but ultimately leads to uncontrolled keratinocyte proliferation and dysfunctional differentiation driven by various inflammatory mediators released in innate and adaptive immune responses, such as IL‐17, IL‐23, TNF‐alpha and Th17 cells.^[^ ^201^ ^]^ Recent findings suggest that close interactions between mediators and cells of the immune system (both innate and adaptive) with keratinocytes and endothelial cells are present in the pathogenetic mechanism of this disease.^[^ ^202^ ^]^ Researchers have examined these mechanisms and proposed several potential biomarkers for psoriasis (presented and summarized in Table 5). These biomarkers mainly come from cell secretion and can be found in the local diseased skin tissue.^[^ ^203^ ^]^ In addition, common inflammation‐related biomarkers are expressed at a different level, either elevated or dropped, from the typical value.^[^ ^204^ ^]^

### 5.3 Vitiligo

Vitiligo is a common chronic autoimmune disease with unknown etiology. It is characterized by melanocyte destruction in the skin, leading to hypopigmentation in the affected area.^[^ ^205^ ^]^ It affects 1% of the world's population with a significant consequence on the sufferer's quality of life, with more than half of cases having an onset of <20 years.^[^ ^206^ ^]^ Vitiligo diagnosis is purely based on visual observation of the white patches that appear on patients’ skin. Classical vitiligo diagnosis is straightforward and can be made in the primary care unit. Atypical presentation of this disease might require expert assessment by a dermatologist.^[^ ^207^ ^]^ Several curative options currently available for vitiligo include topical corticosteroids, calcineurin inhibitors, vitamin D3 analogs, phototherapy, laser therapy, photochemotherapy, antioxidants, and surgical intervention. Depigmentation and camouflage might be considered an option and can be recommended for cosmetic correction, especially if the patient fails repigmentation therapy.^[^ ^208^ ^]^

The problem with vitiligo is that the underlying pathophysiology is not yet clearly understood until recently. Unlike other inflammatory skin disorders, vitiligo lacks obvious inflammatory markers. The visual appearance of this disease can only be observed in active vitiligo. Patients with this condition might have a stable and flaring period at a certain point, which further complicates day‐to‐day vitiligo management. Detailed research has now started to reveal the pathophysiological process of vitiligo. This clearness helps establish valuable biomarkers for diagnosis and prognosis. Moreover, these biomarkers may help assess treatment response across patients, leading to more effective and efficient vitiligo management.^[^ ^209^ ^]^ Proposed biomarkers for vitiligo include microRNA‐211, several proteins, homocysteine amino acids, and some types of clusters of differentiation^[^ ^151^, ^152^, ^153^, ^154^, ^155^, ^156^, ^157^ ^]^ that are summarized in Table 5.

### 5.4 Systemic Lupus Erythematosus (SLE) and Cutaneous Lupus Erythematosus (CLS)

Lupus is a chronic inflammatory autoimmune disease with diverse clinical manifestations due to its effect on multiple organ systems. There are four distinct types of lupus, namely: i) neonatal and pediatric lupus erythematosus (NLE), (ii) discoid lupus erythematosus (DLE); iii) drug‐induced lupus (DIL); and iv) systemic lupus erythematosus (SLE). Although SLE is not specifically a skin disease, it commonly affects the integumentary system. Other organs affected by SLE include the lungs, nervous system, musculoskeletal system, and kidneys.^[^ ^210^ ^]^ If the disease only affects the skin and surrounding connective tissue without systemic clinical manifestation, it is called cutaneous lupus erythematosus (CLS). Clinical presentation includes itching, dyspigmentation and scarring.^[^ ^211^ ^]^ The most common symptoms include malar rash, cutaneous lupus, nonscarring alopecia, leukopenia, arthritis, fever, and Raynaud's phenomenon.^[^ ^212^, ^213^ ^]^

Cutaneous lupus erythematosus (CLS) consists of heterogeneous photodermatoses, commonly classified into three subgroups of acute (ACLE), subacute (SCLE), and chronic (CCLE). Although histopathologically they all present with interface dermatitis (except for tumid lupus and lupus panniculitis) and lupus band reaction, consisting of infiltration of immune cells and deposition of autoantibodies at the dermal‐epidermal junction (DEJ). ACLE is characterized by malar rash, SCLE with nonindurated psoriasiform or annular polycyclic rash, and CCLE, whose most common subtype is discoid lupus erythematosus (DLE), is characterized by erythematous, atrophic and hypopigmented plaques with hyperpigmented borders.^[^ ^214^, ^215^, ^216^ ^]^

The diagnosis of SLE is grounded on a blend of distinctive clinical appearances and positive serology tests. It is often made by classification criteria such as the European League Against Rheumatism (EULAR)/American College of Rheumatology (ACR) 2019, as diagnostic criteria are yet to be available. Even though SLE has an established serological test, the study to find biomarkers for lupus remains ongoing as new bits of knowledge keep unfolding a more robust understanding of the disease's mechanism. For CLE, only a few biomarkers have been identified and incorporated into clinical practice. Specific biomarkers for CLE and SLE may facilitate earlier diagnosis, accurate clinical management, and identification of the personal risk for individual patients as the disease develops.^[^ ^211^, ^213^, ^217^ ^]^

### 5.5 Melanoma

Melanoma is a malignant tumor stemming from melanocytes that mutate into metastatic cells due to the complex interaction of different factors. Melanocytes are located in the basal layer that produces melanin to absorb ultraviolet radiation.^[^ ^218^ ^]^ The critical gene identified in melanoma is the melanocortin 1 receptor (MC1R), which regulates melanin synthesis. Exogenous triggers, endogenous triggers, tumor‐intrinsic factors, and immune‐related factors play significant roles in melanoma development. The incidence varies across the globe, but the trend is that melanoma prevalence has steadily increased over the decades.^[^ ^219^, ^220^, ^221^ ^]^ It is the most aggressive type of skin cancer and accounts for 90% of all skin cancer mortality. Melanoma is not exclusive to the skin and can arise in the eye, meninges, and mucosal surface. Melanoma in the skin is generally referred to as cutaneous melanoma.^[^ ^222^ ^]^ Melanoma prognosis, especially cutaneous melanoma, depends on the depth of invasion/tumor spread at the time of diagnosis. Earlier diagnosis and excision are crucial for patients with this form of malignancy, mainly because of the limited available therapeutics for late‐stage melanoma.^[^ ^223^, ^224^ ^]^

In ≈20–40% of cases, melanoma develops from preceding nevi, while the remaining 60–80% are thought to occur de novo. At the moment, the diagnosis of melanoma is almost entirely performed by a histopathologist through morphological assessment of the tumorous area of the tissue. Melanoma can be diagnosed visually using several tools, including examining superficial spreading melanoma with ABSCE rules, total body photography, and dermoscopy. Several more computerized diagnostic devices are also under investigation for their application.^[^ ^225^, ^226^ ^]^ One of the most commonly used classifications of melanoma was established by Clark based on the depth of invasion by melanoma cells. Level 1: melanoma cells are confined to the epidermis (melanoma in situ); level 2: invasion of single cells or tiny nests of melanoma into the papillary dermis; level 3: melanoma cells “fill and expand” the papillary dermis; level 4: invasion into the reticular dermis; and level 5: invasion into the subcutaneous fat.^[^ ^221^ ^]^

The visual diagnosis of melanoma is limited. The detection of malignant tissue in its early stage could be challenging, and its poor prognosis at the later stage could make patient therapy assessment problematic.^[^ ^227^ ^]^ The American Joint Committee on Cancer (AJCC) staging system provides the first‐line stratification for melanoma‐specific survival using prognostic factors, such as primary tumor thickness, ulceration, mitotic rate and lymph node involvement.^[^ ^168^ ^]^ Better molecular biomarkers and the ability to capture and assess these noninvasively could assist treatment for patients with melanoma, personalized treatment choice, or even help prevent the occurrence in patients at risk.^[^ ^227^, ^228^, ^229^, ^230^ ^]^ Some of the potential biomarkers are listed in Table 5.

### 5.6 Nonmelanoma Skin Cancer

Nonmelanoma skin cancer (NMSC) comprises a group of various cutaneous malignancies, excluding melanoma. NMSC includes squamous cell carcinoma (SCC), basal cell carcinoma (BCC) and Merkel cell carcinoma (MCC).^[^ ^231^ ^]^ BCC is the most common cutaneous cancer, accounting for 80% of all skin cancers. They usually occur in the head and neck region. BCC is locally destructive but rarely metastasizes, causing significant morbidity to patients. SCC is the second most common skin cancer that develops in patients with lighter skin. Clinically and histologically, it can be classified into low‐risk and high‐risk. The clinical manifestation of SCC varies from indolent behavior with slow growth to aggressive tumors showing invasive properties and high spreading toward distant sites. MCC, also known as primary cutaneous neuroendocrine carcinoma, is a rare skin malignancy characterized by aggressive clinical behavior. MCC is a poorly differentiated neuroendocrine carcinoma that lacks a recognized benign or dysplastic precursor. A clinical manifestation usually presents as a solitary, painless, red or violaceous intracutaneous nodule rapidly growing on the sun‐exposed skin of elderly, fair‐skinned individuals.^[^ ^231^, ^232^, ^233^ ^]^

Populations with high UV radiation exposure and fair skin are susceptible to NMSC. In addition, genetic predisposition, immunosuppressant uptake, immunocompromised status, arsenic exposure, age, and sex are other risk factors that contribute to the occurrence of this disease.^[^ ^232^, ^234^ ^]^ For MCC, infectious viruses such as human papillomavirus (HPV), Epstein‐Barr virus (EBV), and Merkel cell polyomavirus (MCPyV) may trigger the development of this type of NMSC with varying mechanisms.^[^ ^228^ ^]^ HPV can produce an oncoprotein that can integrate with the host genome,^[^ ^235^, ^236^ ^]^ whereas EBV infection can drive both genetic and epigenetic changes in the keratinocyte genome,^[^ ^237^ ^]^ and MCPyV integrates its DNA into the host cell and triggers the production of carcinogenic proteins, large T‐antigen (LTAg) and small t‐antigen (STAg).^[^ ^228^ ^]^

Diagnosis of NMSC is performed through a physical examination to find and identify lesions and then confirmed by skin biopsy histopathological analysis. As an alternative to direct invasive biopsy, techniques such as dermoscopy, confocal microscopy, cross‐polarized light and fluorescence photography, and optical coherence tomography (OCT) combined with high‐frequency ultrasound can be used to preexamine suspected lesions and avoid unnecessary intervention. Multiphoton microscopy and Raman spectroscopy are proposed to be used for the same purposes.^[^ ^238^ ^]^ Some genetic and serological biomarkers have been proposed and studied extensively to assist in the pathological‐heavy diagnosis of NMCS.^[^ ^171^ ^]^ Despite its significant advantages, the application of these biomarkers in NMCS has not yet been established. NMCS is significantly associated with UV exposure, and the prognosis is known to be related to overexpression of tumor‐associated antigens (TAAs). Establishing UV exposure‐related biomarkers and autoantibodies secreted in response to TAAs could be very useful in stratifying patient risk and ensuring future routine cancer screening.^[^ ^171^, ^239^, ^240^ ^]^

### 5.7 Periodontitis

Periodontitis is a multifactorial oral disease with microbial dental plaques as the originator. It is an endemic infectious disease that causes inflammation of the tissue surrounding the teeth. It occurs in ≈50% of the population, and severe periodontitis can be found in 5% to 15% of most populations worldwide. Periodontitis is considered one of the most important global oral health burdens.^[^ ^241^ ^]^ Smoking, poor oral hygiene, hormonal change, preexisting diabetes mellitus, medication, stress, age, and hereditary are risk factors for periodontal disease.^[^ ^242^ ^]^ Untreated periodontitis is closely associated with systemic diseases, such as cardiovascular disorder, diabetes, Alzheimer's disease and several other conditions due to the bacteria's accessible inflamed periodontal pocket, which can cause metastatic infection and inflammation.^[^ ^243^ ^]^

Pathogenic bacteria take over the periodontal sulcus/pocket during disease onset and progression to form subgingival oral biofilms. They primarily attach to the tooth root surface, triggering inflammation in the periodontal tissues. In severe cases, the obliteration of periodontal tissues leads to gradual attachment loss advancing to tooth loss. Periodontitis is currently diagnosed using radiography and clinical appraisal. Probing pocket depth (PD), bleeding on probing (BOP), and clinical attachment level (CAL) are used in the assessment. The disease can be treated, but periodontal health needs to be constantly observed after initial treatment and resolution. In some individuals, the condition may reoccur and develop without apparent symptoms. With no reliable prognostic markers available, patients’ treatment needs will be identified only after tissue destruction is evident.^[^ ^244^, ^245^ ^]^

### 5.8 Glaucoma

Glaucoma, a group of optic neuropathies, is one of the preeminent causes of irreversible blindness worldwide. The most common form of this condition is primary open‐angle glaucoma (POAG), with a reported prevalence of ≈3% in 2013 in the population aged between 40–80 years. It is commonly viewed as a neurodegenerative disease with a multifactorial origin. It is typically defined by a chronic, slowly progressive optic neuropathy with characteristic patterns of optic nerve damage and visual field loss. Other than increasing intraocular pressure (IOP), older age, race, thin central cornea and low corneal hysteresis, family history, and myopia are important risk factors for POAG. Although glaucomatous optic neuropathy has diverse subtypes, all result in irreparable visual field loss and blindness.^[^ ^246^, ^247^ ^]^

Current screening techniques, such as elevated IOP and perimetry‐based examination, have poor sensitivity and cannot diagnose the early onset of POAG. Approximately 30–50% of glaucoma patients have an IOP of less than 22 mmHg, making elevated IOP less reliable for diagnosis. It has not been determined whether thin central corneal tissue (CCT) causes underestimation of IOP or whether thin central corneal tissue (CCT) itself could be a biomarker for glaucoma as another independent risk factor.^[^ ^248^ ^]^ Tests used for glaucoma screening include tonometry, dilated optic disc evaluation, slit‐lamp, gonioscopy, and visual field examination. Screening using automated perimetry for glaucomatous visual field defects lacks resolution to detect early POAG. The issue with low resolution visual field observation is that 35% of the retinal ganglion cells can be lost before any visual field impairment can be observed.^[^ ^249^ ^]^ Another imaging technique to detect early glaucoma based on anatomical structures is OCT (retinal nerve fiber layer [RNFL], optic nerve head [ONH], and macula parameters) and OCT angiography. Early diagnosis using biomarkers has been proposed for better disease management and the decision of therapeutic options. Tear fluid is a potential rich source of biomarkers, conjunctival tissue, and aqueous humor.^[^ ^76^ ^]^

## 6 Connecting the Dots: Microneedle Meets Biomarkers

Employing MAP‐based biomarker detection could enable a shift from visual scoring‐based diagnosis dependency to a more quantitative lab‐based assisted diagnosis approach. The diseases discussed in the previous section rely heavily on clinical scoring, where the features might not develop until the diseases reach a particular stage, hindering earlier screening and resulting in poor disease prognosis. Earlier diagnosis is critical in conditions such as melanoma, as it can increase the survival rate.^[^ ^223^ ^]^ A reliable prognosis assessment plays a significant role in AD management.^[^ ^4^, ^6^ ^]^ In addition, biomarker‐based detection can help differentiate diseases such as AD versus psoriasis cases.^[^ ^4^ ^]^ Other diseases usually accompanied by comorbidities, such as CLE, would also benefit from biomarker‐based diagnosis, as some lesions might not fall into the typical plaque type or show a minimal pathology.^[^ ^15^ ^]^

AD management is a good example in which the demand for applying biomarker‐based diagnosis is high. AD is a heterogeneous disease with >3 different phenotypes. Although blood biomarkers are generally preferred, the interest in skin biomarkers is still high.^[^ ^4^ ^]^ Biomarkers such as thymus and activation‐regulated chemokines, macrophage‐derived chemokines, e‐selectin, eotaxin‐3, adipokines (adiponectin and resistin) and immunoglobulin E have been proposed as biomarkers, and those marker molecules can be isolated from serum.^[^ ^136^, ^137^, ^138^, ^139^, ^140^, ^141^ ^]^ A proteomics analysis from ISF samples collected using hollow microneedles demonstrated the presence of immunoglobulins, including IgE, in the skin.^[^ ^45^ ^]^ Hypothetically speaking, using MAP arrays might allow minimally invasive monitoring of IgE elevation in skin tissue.

**Figure 11:**
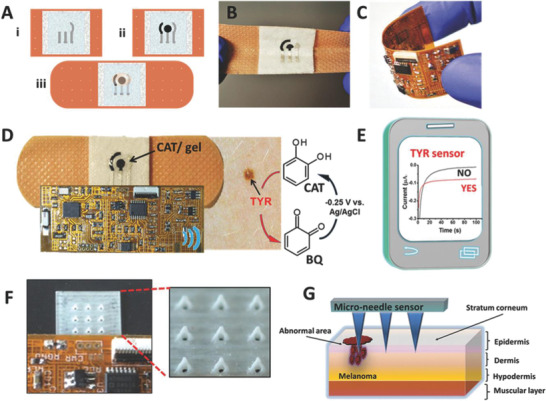
Fabrication of integrated MAPs with sensors and a built‐in processor for tyrosinase detection A), representative images of the bending of wearable bandage sensor B), the flexibility of the approach developed C), the procedure of tyrosinase sensing D), illustrative graph describing that amperometric data is wirelessly transmitted to the device E), MAPs combined with soft flexible electronics F), and the application of MAPs to detect tyrosinase melanoma biomarkers G). Reproduced with permission.^[^ ^132^ ^]^ Copyright 2018, Wiley‐VCH GmbH.

For more clinically relevant research, we can take melanoma as the first example. It is diagnosed using mainly visual scoring and identification of biomarkers involving time‐consuming and complex procedures. Although many biomarkers have been proposed as prognostic biomarkers, such as lactate dehydrogenase and tyrosinase, which are easier to perform as regular laboratory tests,^[^ ^17^ ^]^ many of them cannot be used for earlier diagnosis using the existing biomatrix, sampling method and analytical method. However, at this moment, the researcher also knows that tyrosinase accumulates in the skin as it is overexpressed by malignant cells,^[^ ^250^ ^]^ which can serve as a helpful marker, especially for initial screening. Ciui et al. (2018) proposed their invention utilizing MAPs integrated with sensors and a built‐in processor for tyrosinase detection (**Figure** **11**). The whole device takes the form of a band aid with compact size and wireless connectivity for data visualization and interpretation.^[^ ^132^ ^]^

**Figure 12:**
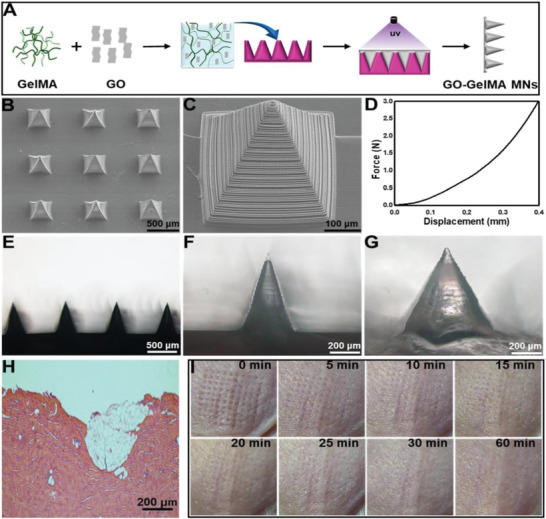
Illustrative images of preparation of MAPs A), SEM images of MAPs using different magnifications B,C), mechanical properties of MAPs D), light microscope images of MAPs using different magnifications E,F), illustrative images of MAPs following 30 min sampling process in 1% agarose G), histological images of porcine skin following the administration of MAPs, stained using H&E H), and representative of skin of mouse at different time points following the application of MAPs I). Reproduced with permission.^[^ ^251^ ^]^ Copyright 2022, American Chemical Society.

Another recent case is in psoriasis diagnosis. Qiao et al. (2022) demonstrated the versatility of MAPs to sample genetic material to aid in the diagnosis of psoriasis (**Figure** **12**). MicroRNAs are expressed in most psoriatic tissues and can be found in peripheral blood and skin interstitial fluid. This group proposed the use of graphene oxide‐functionalized gelatin methacryloyl‐based HFMAPs. They can capture and detect microRNA‐related psoriasis, including miR‐21, miR‐141 and miR‐155, from the psoriatic mouse model after 5 min of application time. Using this design, microRNA can be detected on‐needle and in extraction solution (off‐site analysis), showing the flexibility of hydrogel‐forming MAPs in terms of analytical method suitability. On the safety side, their work shows that the microperforations created by the MAPs were healed in under 60 min.^[^ ^251^ ^]^

A more generic study reported by Kim et al. (2022) highlights the potential use of microsampling using MAPs made from hyaluronic acid for transcriptome analysis of human skin. Their results indicated a correlation between different biomarkers at the mRNA expression level and patient age. These results can be helpful in different pathological conditions, such as psoriasis, vitiligo and atopic dermatitis.^[^ ^252^ ^]^ Another generic approach was made by Ng et al. (2015), where they used coated solid MAPs in conjunction with a blotting method for skin disease biomarkers. They demonstrate that PLA‐based solid MAPs produced through simple micromoulding can be functionalized with different capture antibodies. This approach allows multiplex analysis of different biomarkers, such as TNF‐*α*, IL‐6 and IL‐1*α*, by coating different groups of needles in the same array with different capture antibodies and performing a blotting assay for each biomarker.^[^ ^126^ ^]^

Another critical consideration is that signal normalization to known abundance biomarkers is needed. This normalization allows the consistent measurements and interpretations of ex situ analysis carried out after MAP application.^[^ ^253^, ^254^ ^]^ Normalization is important regarding the type of MAPs used in ISF sampling, as it has been highlighted for both hydrogel‐forming and solid MAP extraction.^[^ ^30^, ^253^ ^]^ The choice of analyte for normalization needs to be considered carefully, as the concentrations of these markers could change depending on the physiological state of the subject (such as healthy versus diseased subjects). In addition, the mechanism of ISF sampling should not alter the concentration of the analyte used as the internal standard for normalization.^[^ ^30^ ^]^

Despite all the challenges and limitations, these examples show that MAP technology could potentially: 1) enable rapid and minimally invasive ISF‐based diagnosis; 2) expand the range of the utilization of biomarkers for early detection and screening of localized disorders; 3) expand the range of clinically relevant biomarkers that are usually not accessible (e.g., available mainly on diseased tissue); 4) exploit MAP‐mediated diagnosis in conjunction with different established bioanalytical techniques; 5) allow specific and targeted biomarker capture and detection; and 6) with sufficient technology, building wearable devices can be the future of localized disease screening and monitoring.

One question might arise: if MAP technology is beneficial for localized disease diagnosis and monitoring, especially for external organs, why are there only a few studies focusing on this application? Answering this question is like answering the old riddle, which one comes first, the egg or the chicken? MAPs technology is still evolving as scientists refine the design to make the procedure mediated by MAPs more robust, sensitive, and selective. On the other hand, the researcher also keeps trying to push clinical biomarkers based on localized tissue fluid for the external organ condition, but the availability of a consistent sampling method largely hinders it. In our proposal, we suggest that the progress of both MAP‐mediated diagnosis and the unfolding of clinically relevant biomarkers for external organ conditions are parallel work that does not exactly precede one another. However, suppose researchers start to adopt the available prototype to aid the biomarker search in various external organ disorders. In that case, the progress could be accelerated as the technology, and the clinical information could mature simultaneously.

## 7 Future Directions and Conclusion

External human body parts are continuously exposed to various environmental factors, such as infectious microbes, toxins, chemicals, UV light and radiation, which can have pathophysiological consequences. Recent advances in microstructures such as microneedles and microsensors could allow the early and effective diagnosis of localized disease conditions. The vast range of various microneedle systems provides flexibility in designing the right system with specific diagnosis requirements with simple extraction of biosamples to built‐in disease marker sensing tools. Thus, there is enormous potential to achieve an MAP‐based product for collecting the biofluid from the respective tissue site as far as enough volume is collected and separated for external organ disease detection devices with high specificity.

Recently, there has been growing interest in the development of MAP‐based built‐in biosensing devices. The possibility of combined lab‐on‐a‐chip MAP devices is an especially exciting one since it could accelerate the diagnosis by circumventing the necessity of centralized test facilities. The improvement of MAP‐based biosensor detection for analytes of interest could be revolutionary within the diagnostics sector. However, some essential criteria to fulfil for this early‐stage proof of concept research work to become clinically adopted, in particular the high reproducibility and stability of MAP systems. Early‐stage diagnostics are always highly attractive but often limited by an undetectable/insignificant change in potential biomarkers, creating another challenge requiring a lower detection limit. Therefore, current MAP research is needed to improve the detection limit through a combination of optimized fluid collection, biosensors, detection devices, and signal processing algorithms.

As mentioned previously, one of the major challenges that need to be addressed regarding microneedle arrays for fluid extraction is how to recover enough volume in a short amount of time. Up to 16 µL of ISF recovery in 1–2 hours has been reported in the attempt to sample ISF using hollow MAP arrays.^[^ ^38^ ^]^ The recovered ISF volume was very small compared to the milliliter volume that could be collected from blood sampling. Judging from the current progress in the research thus far, the number of analyses that can be carried out per ISF sample collected will be very limited. Hence, ISF‐based diagnosis/monitoring should only be considered where the benefit of carrying out the analysis should weigh more than the burden of performing the procedure. Currently, this problem can be overcome by pooling the samples to collect a sufficient amount of fluid for analysis.^[^ ^38^, ^255^ ^]^

Biomarker analysis can only be as good as its limit of detection. This problem has specifically impaired the use of MAP‐based devices for diagnosis and monitoring, as its sample volume is already small enough. The advancement of the bioanalytical method will aid the problem, especially for ex situ analysis.^[^ ^256^ ^]^ Meanwhile, for onboard analysis, improvement of energy‐efficient microchip processors and biosensors^[^ ^257^ ^]^ was essential to push the technology further down in the translational pipeline.^[^ ^258^ ^]^ Another obstacle for clinical translation is the large‐scale fabrication of MAP devices at cost‐effective ranges.

A large amount of data is needed to verify the clinical relevancies of MAP‐based sampling and detection. ISF sampling through MAP devices has proven safe in small‐scale trials. After stainless steel MAP application, the skin can recover within a day.^[^ ^30^ ^]^ Other studies have demonstrated that several inflammatory and immunologic biomarkers do not change after polymeric hydrogel‐forming MAP application.^[^ ^103^, ^104^ ^]^ However, it is essential to highlight that the type of MAPs might affect the type and magnitude of biomarkers collected, as demonstrated by a proteomic analysis study comparing the pool of biomarkers extracted from different types of superswelling hydrogels by Laszlo et al. (2021).^[^ ^26^ ^]^ As per US FDA guidelines, nondrug‐loaded MAP devices intended for diagnosis can be registered as a device.^[^ ^259^ ^]^ The approval of MAP‐based devices should not be as tedious as the drug route, further shortening its translational process.

Each technology has limitations, including MAP‐based diagnostic devices. Their limitations mainly derive from the inherent characteristics of MAP arrays and sensing units. Hydrogel‐based arrays are limited by their ability to swell, the charge of the hydrogel network and the hydrogel mesh size, as they determine the type and magnitude of molecules transported through the hydrogel network. A charged polymer is often employed to form a highly swollen hydrogel, limiting the pool of molecules that can be extracted from the skin. This property could be used as an advantage, where extraction of specific charged molecules is favored^[^ ^124^ ^]^ but simultaneously limits its generic use. Dissolving and solid MAPs can only be used to perforate the skin, and another accessory part is necessary to collect and pool the extracted ISF. From this perspective, hollow and porous MAP arrays seem to be more promising options.

More limitations come from the sensing and quantification of the bioanalytes. Off‐site analysis of ISF is ideal, as it allows analysis with high specificity and sensitivity (e.g., ELISA, LC–MS/MS), but the process is time‐consuming and requires advanced instrumentation.^[^ ^123^, ^124^, ^125^, ^126^, ^128^ ^]^ On‐board analysis is limited by the type of detection modality used or the type of antibody used, limiting their use to one or two analytes per measurement.^[^ ^27^, ^118^, ^130^, ^132^, ^133^ ^]^ Paper‐based analysis is simple and can be used with hydrogel‐forming or hollow/porous MAPs, but it can only be used for qualitative to semiquantitative analysis.^[^ ^260^ ^]^ Hollow and porous MAPs are more flexible, as they allow direct fluid sampling. Integration of microfluidics chips can further enhance the range of applications for these types of arrays. Regarding the sensing modality, most prototypes employ electrochemical sensors based on enzymatic reactions to produce electrical signals. Electrochemical sensing is often limited to small molecule detection (such as glucose),^[^ ^130^, ^133^ ^]^ although protein biomarker detection has been demonstrated by using immobilized capture antibodies.^[^ ^27^ ^]^

A joint consortium between academia, industry, government funding, and regulatory bodies could accelerate the process to overcome the bridge between early research concepts and clinical products for patient benefit. The problems discussed above need an interdisciplinary approach to address, as expertise in MAP design and formulation, sensors, microprocessors and bioanalysis are not monopolized by a single discipline. Physician inputs are highly needed, and patient response is critical for the technology to reach maturity. Further MAP‐based point‐of‐care (POC) diagnosis potential could be realized by replacing the current laborious testing methods.

To conclude, ISF, GCF, and AH are promising biomatrices for different types of molecules with diagnostic value but are currently underutilized due to the limitation of a minimally invasive sampling method. MAP devices open the opportunity to explore ISF‐based diagnosis for external organ disease where detecting biomarkers from surrounding tissue is more clinically relevant than measurement based on systemic fluids such as serum and urine. Multiple proof‐of‐concept studies have shown that MAP devices can be used to sample ISF directly or capture biomarkers from the underlying tissue, for example, in skin, eyes and oral cavity. The development of these technologies to sample the ISF and report data in real time has the potential to create point‐of‐care diagnostic assays and systems that will facilitate clinical decision making and ultimately outcomes for patients.

## Conflict of Interest

R.F.D. is an inventor of patents that have been licensed to companies developing microneedle‐based products and is a paid advisor to companies developing microneedle‐based products. The resulting potential conflict of interest has been disclosed and is managed by Queen's University Belfast. The companies had no role in the design of the review, in the collection, analyses, or interpretation of the literature, in the writing of the manuscript or in the decision to publish the review. After initial online publication, the name of the university in the third affiliation was changed to “Hasanuddin University” on February 17, 2023.
